# Chaotic opposition learning with mirror reflection and worst individual disturbance grey wolf optimizer for continuous global numerical optimization

**DOI:** 10.1038/s41598-024-55040-6

**Published:** 2024-02-26

**Authors:** Oluwatayomi Rereloluwa Adegboye, Afi Kekeli Feda, Opeoluwa Seun Ojekemi, Ephraim Bonah Agyekum, Abdelazim G. Hussien, Salah Kamel

**Affiliations:** 1 Management Information Systems, University of Mediterranean Karpasia, Mersin-10, Turkey; 2 Management Information System Department, European University of Lefke, Mersin-10, Turkey; 3 Engineering Management, University of Mediterranean Karpasia, Mersin-10, Turkey; 4 Department of Nuclear and Renewable Energy, Ural Federal University Named After the First President of Russia Boris Yeltsin, 19 Mira Street, Yekaterinburg, Russia 620002; 5 Department of Computer and Information Science, Linköping University, Linköping, Sweden; 6 Faculty of Science, Fayoum University, El Faiyûm, Egypt; 7 Applied Science Research Center, Applied Science Private University, Amman, 11931 Jordan; 8 MEU Research Unit, Middle East University, Amman, 11831 Jordan; 9 Electrical Engineering Department, Faculty of Engineering, Aswan University, Aswan, 81542 Egypt

**Keywords:** Computational science, Computer science, Information technology, Software

## Abstract

The effective meta-heuristic technique known as the grey wolf optimizer (GWO) has shown its proficiency. However, due to its reliance on the alpha wolf for guiding the position updates of search agents, the risk of being trapped in a local optimal solution is notable. Furthermore, during stagnation, the convergence of other search wolves towards this alpha wolf results in a lack of diversity within the population. Hence, this research introduces an enhanced version of the GWO algorithm designed to tackle numerical optimization challenges. The enhanced GWO incorporates innovative approaches such as Chaotic Opposition Learning (COL), Mirror Reflection Strategy (MRS), and Worst Individual Disturbance (WID), and it’s called CMWGWO. MRS, in particular, empowers certain wolves to extend their exploration range, thus enhancing the global search capability. By employing COL, diversification is intensified, leading to reduced solution stagnation, improved search precision, and an overall boost in accuracy. The integration of WID fosters more effective information exchange between the least and most successful wolves, facilitating a successful exit from local optima and significantly enhancing exploration potential. To validate the superiority of CMWGWO, a comprehensive evaluation is conducted. A wide array of 23 benchmark functions, spanning dimensions from 30 to 500, ten CEC19 functions, and three engineering problems are used for experimentation. The empirical findings vividly demonstrate that CMWGWO surpasses the original GWO in terms of convergence accuracy and robust optimization capabilities.

## Introduction

Optimization is described as the process of determining the most suitable values for the parameters of a problem with the goal to obtain the ideal solution^1^. Optimization Algorithms have gained acknowledgment as effective instruments for improving various types of single-objective, multi-objective, and many-objective problems^2^. The effectiveness of these algorithms has resulted in the creation of a large number of swarm intelligence algorithms and their extensive use in numerous applications across numerous fields^3^. Swarm intelligence algorithms are developed by studying the interactions of self-organized living beings in nature and are a subset of Metaheuristic Algorithms (MAs)^4^. Examples of recent MAs include Gannet Optimization Algorithm (GOA)^5^, African Vultures Optimization Algorithm (AVOA)^6^, Material Generation Algorithm (MGA)^7^, Beluga Whale Optimization (BWO)^8^, Archimedes Optimization Algorithm (AOA)^9^, Artificial Gorilla Troops Optimizer (GTO)^10^, Dandelion Optimizer (DO)^11^, Golden Eagle Optimizer (GEO)^12^, Chaos Game Optimization (CGO)^13^, Fire Hawk Optimizer (FHO)^14^ and Honey Badger Algorithm (HBA)^15^. It is also worthwhile to explore certain modified algorithms that exhibit exceptional performance, such as Modified Social Group Optimization (MSGO)^16^, Chaotic Vortex Search Algorithm (VSA)^17^, Modified Marine Predators Algorithm (MMPA)^18^, and Hybrid Binary Dwarf Mongoose Optimization Algorithm (BDMSAO)^19^. They have found practical applications in various domains, including parameter identification^20^, feature selection^21,22^, Antenna Optimization^23^, Image Segmentation^24,25^, demand prediction^26^, Reliability-Based Design^27,28^, constrained optimization problems^21,22^. These algorithms, however, share several challenges, such as a propensity to get trapped in local optimal solutions, sluggish convergence rate, and limited precision in identifying the optimal solution^29^.

The Grey Wolf Optimizer (GWO) is a Swarm intelligence metaheuristic algorithm developed by Mirjalili et al. that emulates the leadership structure and hunting behaviour of grey wolves in the wild^30^. The GWO algorithm has been successfully used to address different optimization problems, including numerical optimization^31^, feature subset selection^32^, engineering design^33^, image analysis^34^, and other real-world applications^35^. Researchers have attempted to improve the original GWO by creating various variants, which can be categorized into two groups. The first group focuses on implementing distinct optimization strategies to overcome GWO's limitations. The second group includes variants that combine GWO with other algorithms to enhance its optimization capabilities by leveraging the advantages of these combined algorithms.

In the first group, Nadimi-Shahraki et al.^36^ introduced the Improved Grey Wolf Optimizer (I-GWO) that incorporates a novel movement strategy called dimension learning-based hunting (DLH) search strategy, modelled after the solitary hunting tactics used by wolves in the wild. DLH establishes wolf neighbourhoods in a way to facilitate the exchange of neighboring information among them. The incorporation of dimension learning in the DLH search strategy improves the equilibrium between local and global search and diversity preservation in the optimization process. The proposed I-GWO algorithm's efficacy was assessed using the CEC 2018 test set and four real-world problems. I-GWO is contrasted across many tests to six other algorithms. Friedman and Mean Absolute Error (MAE) statistical tests are also used to assess the results. In comparison to the algorithms employed in the studies, the I-GWO algorithm was highly efficient and frequently outstanding. Mirjalili et al. proposed a Multi-Objective Grey Wolf Optimizer (MOGWO) to address multi-objective problems’ optimization^37^. For that purpose, a fixed-sized external archive was incorporated into the GWO, serving as a repository to store and retrieve the best solutions. The incorporated archive influences the definition of social ranking and the emulation of grey wolves’ hunting patterns in multi-objective search areas. To assess its performance, the novel MOGWO was evaluated on ten multi-objective standard problems and benchmarked against two other popular MAs. The outputs of the assessments indicate that the MOGWO algorithm surpassed the other MAs under consideration in terms of performance. Bansal and Singh suggested an improved grey wolf optimizer to enhance the exploration and exploitation capabilities of the traditional GWO^38^. Opposition-based learning (OBL) and the explorative equation were used to make this improvement. The explorative equation contributed to improving GWO's capacity for exploration. The OBL sped up convergence and prevented the GWO from stagnating. 23 popular standard functions were used to evaluate the suggested IGWO. The results have been contrasted against some recent GWO versions along with additional well-known MAs. The results confirmed that the IGWO has better exploration capabilities while yet retaining an excellent speed of convergence. Meidani et al. presented another variant called Adaptive GWO (AGWO) that tackles the non-automated variable adjustment and absence of precise stopping conditions that frequently result in wasteful consumption of computing resources^39^. The optimization process was carried out by incorporating an adaptive calibration of the intensification/diversification variables depending on the fitness records of the potential solutions. A satisfactory optimal solution can be reached by AGWO within a brief period by regulating the stopping criteria depending on the importance of fitness increase in the optimization. Through a comprehensive comparative study, they demonstrated that AGWO is significantly more efficient than the original GWO and a number of GWO variations that were already in use. AGWO achieved this by lowering the number of iterations necessary to arrive at similar solutions to those of GWO. Lei et al. introduced Levy Flight to the GWO (LFGWO) to tackle the challenges of premature convergence and inadequate results^40^. By conducting experiments with eight common algorithms and 23 common benchmark functions from CEC 2005, the overall performance of LFGWO was assessed. The findings showed that LFGWO performs better than the competing algorithms. Gutpa and Deep introduced a revised RWGWO employing a random walk in an effort to enhance the grey wolf's search capabilities^41^. The algorithm's performance is demonstrated by comparing it with GWO and other advanced algorithms using IEEE CEC 2014 benchmark problems. To gauge the effect of enhancing the leaders in the proposed algorithm, a non-parametric test, Wilcoxon, and Performance Index Analysis were used to analyze outcomes. The findings show that the suggested algorithm offers grey wolves greater leadership when searching for prey. Nasrabadi et al. introduced parallelism and opposition-based learning methods in an attempt to enhance the basic GWO’s outcomes^42^. The setup and execution of the revised method on renowned benchmark functions yielded results that showed improvements in convergence and accuracy.

In the second group also, noteworthy outcomes were achieved by researchers. By integrating the Elephant Herding Optimization (EHO) algorithm with the Grey Wolf Optimizer (GWO), the exploitation and exploration performances, as well as the speed of convergence, of the GWO were significantly enhanced by Hoseini et al.^43^. To confirm the effectiveness of the proposed Grey Wolf Optimizer Elephant Herding Optimization (GWOEHO), a set of twenty-three benchmark functions and six engineering problems were employed for testing. The performance of GWOEHO was compared to that of the GWO and EHO, along with several other popular MAs. The statistical analysis using Wilcoxon's rank-sum test demonstrates that GWOEHO consistently performed better than the other algorithms in the majority of function minimization tasks. With the merging of Particle Swarm Optimization and Grey Wolf Optimizer, Singh and Singh formed a Hybrid Particle Swarm Optimization and Grey Wolf Optimizer (HPSOGWO)^44^. The major goal was to increase the exploration and exploitation capacities of the two algorithms to boost their strengths. A few unimodal, multimodal, and fixed-dimension multimodal testing functions were employed to evaluate the effectiveness and efficacy of the HPSOGWO. The hybrid algorithm greatly exceeded the PSO and GWO versions in terms of outcome effectiveness, robustness, speed, and capacity to reach the global optimum. Zhao et al. presented another hybrid variant of the grey wolf optimizer that integrates opposition-based learning, reinforcement learning, and sine cosine search strategy^45^. The novel algorithm was employed for scheduling and resource allocation. To validate its effectiveness, six sets of realistic data related to space debris tracking were selected. The proposed algorithm's performance was evaluated in comparison with that of other algorithms. The experimental results demonstrate that the proposed algorithm successfully tackles the resource allocation and scheduling challenges associated with space debris tracking. Fadheel et al. proposed the Sparrow Search Algorithm-Grey Wolf Optimizer (SSAGWO), which is designed for the precise tuning of controllers used in frequency regulation^46^. The authors succeeded in enhancing the original algorithms' capabilities for exploration and exploitation. SSAGWO was applied to regulate frequency in a two-area Hybrid Power System (HPS) simulated in Simulink. To validate the efficacy of the hybrid SSAGWO in controlling the frequency of the HPS model, its performance is first evaluated using common benchmark functions. The results clearly demonstrate that the hybrid SSAGWO significantly outperforms other state-of-the-art algorithms. J and Priya also recently introduced another variant of the GWO, known as the Hybrid Grey Wolf and Improved Particle Swarm Optimization Algorithm with Adaptive Inertial Weight-based multi-dimensional Learning Strategy (HGWIPSOA) to improve the precision and efficiency of task scheduling and resource allocation for Virtual Machines (VMs) in cloud environments^47^. The algorithm begins by integrating the Grey Wolf Optimization Algorithm (GWOA) into the Particle Swarm Optimization (PSO), treating the highest fitness particle as the alpha wolf search agent. This integration effectively achieves the task allocation objective for VMs. Additionally, the suggested method combines PSO with chaos, Adaptive Inertial Weight, and Dimensional Learning. These additional features rely on the best experiences decided by particles to support efficient Load Balancing, with the goals of preventing early convergence, improving convergence pace, and enhancing overall search capabilities. The HGWIPSOA 's higher performance was demonstrated in simulation trials, and significant advancements were seen. Large tasks were presented in the cloud environment, improvements are constantly seen, putting the proposed HGWIPSOA on a level with benchmarked Load Balancing methods.

While these improved GWO variants address the limitations of GWO to a certain degree, there remains potential for further enhancement, especially in terms of population diversity, which affects convergence speed, precision, and vulnerability to getting trapped in local optima. The original Grey Wolf Optimizer operates by utilizing the three most successful wolves in each iteration to guide the search process, resulting in significant convergence towards these wolves. However, there are instances where these leading wolves become trapped in local extreme points or fail to locate the global optimal solution, particularly in problems with multiple locally optimal solutions. Consequently, when the leading wolves encounter local optima, other individuals in the population also become susceptible to local extremes. This phenomenon contributes to a decrease in population diversity as the wolves converge toward the leaders. Although authors have demonstrated significant progress in improving the conventional Grey Wolf Optimizer (GWO) through various enhancement techniques and hybridization approaches, the literature review reveals a lack of consideration for utilizing physics-inspired techniques and leveraging information from the worst wolf to escape local optima and address population diversity. These fundamental issues pose challenges to the traditional GWO and serve as the primary motivation for this research. This research aims to address these issues by introducing a novel approach called Chaotic Opposition Learning with Mirror Reflection and Worst Individual Disturbance Grey Wolf Optimizer (CMWGWO). The CMWGWO incorporates three distinct search strategies with unique characteristics to generate and enhance candidate solutions. One of these strategies is Chaotic Opposition learning, which draws inspiration from the concept that the opposite of a current solution may yield a superior solution. By leveraging this strategy, population diversity is improved throughout the search space, facilitating better escape from local optima. However, since opposition learning may lead to suboptimal trapping, chaotic randomness is introduced through chaotic Map functions to introduce more randomness to the opposition solution, thereby enabling the algorithm to discover additional potential solutions. Additionally, the Mirror Reflection Strategy which is a physics-inspired phenomenon is integrated into the updating process to amplify population exploration and expand the search space. This enables the population to broaden their search range and approach the optimal solution more closely. Furthermore, the Worst individual disturbance strategy is implemented to disrupt the dominance of the leading wolves. This approach allows wolves to update their positions based on the worst-performing wolf with a certain probability, enabling them to break free from local optima even when the three best-performing wolves are trapped. It also promotes better trapping of prey. By incorporating this strategy, the proposed CMWGWO achieves a balance between exploration and exploitation by exchanging and merging information between the best and worst wolves, ultimately leading to the discovery of the global optimum. CMWGWO distinguishes itself from the recently proposed state of art optimizers such as Bonbo Optimizer (BO)^48^, Quantum-based Avian Navigation optimizer Algorithm (QANA)^49^, and Starling Murmuration Optimizer (SMO)^50^ by combining the hunting hierarchy of GWO, Chaotic Opposition learning, Mirror Reflection Strategy, and Worst Individual Disturbance for enhanced exploration and escape from local optima. BO relies on a fission–fusion social strategy inspired by Bonobos, QANA integrates quantum principles for navigation, and SMO emphasizes dynamic multi-flock construction for effective exploration. Each algorithm has unique features tailored to specific inspirations, making them suitable for different optimization challenges. The research presented in this study contributes in the following ways:

1. A novel Grey Wolf Optimizer (GWO) approach is introduced, incorporating Chaotic Opposition learning, Mirror Reflection Strategy, and Worst Individual Disturbance. This innovative GWO variant is specifically designed for Global Numerical Optimization problems.
2. By incorporating Chaotic Opposition learning into GWO, the algorithm mitigates stagnation and enhances diversification, leading to improved solution accuracy.
3. The integration of the Mirror Reflection Strategy into the GWO updating process amplifies population exploration and expands the search space. This enables the algorithm to explore a wider range of potential solutions.
4. The proposed worst individual disturbance strategy reduces the probability of the algorithm getting stuck in local optima. By exchanging information between the best and worst wolves, it enhances population diversity and improves the algorithm’s ability to trap prey.
5. The performance of the proposed algorithm is thoroughly evaluated by comparing it to nine other algorithms across twenty-three test functions. This evaluation provides insights into its effectiveness and efficiency.
6. In addition to numerical optimization problems, the proposed algorithm is also evaluated on three engineering design issues, demonstrating its applicability and effectiveness in solving practical problems.

The subsequent sections of this paper are organized as follows: “Grey wolf optimizer (GWO)” section provides an introduction to the background of GWO. In “Proposed CMWGWO” section, the proposed algorithm’s mechanism is explained and the proposed CMWGWO is presented. The complexity of the new CMWGWO is discussed in “Computational complexity of CMWGWO” section, The experimental results are discussed and displayed in “Experiments and result analysis” section. Lastly, “Conclusion” section concludes the paper and outlines future research directions.

## Grey wolf optimizer (GWO)

**Figure 1:**
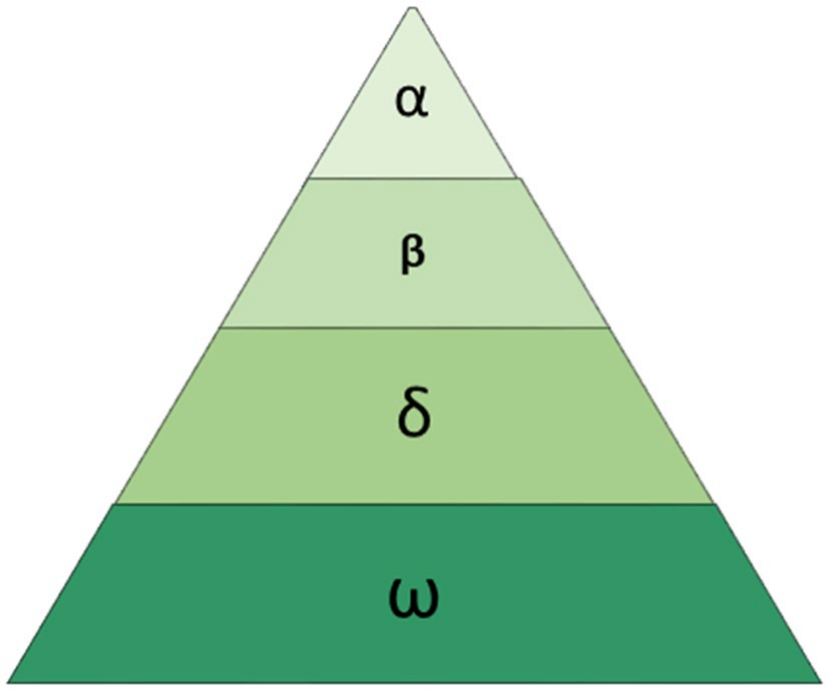
Hierarchical model of GWO.

GWO, an optimization algorithm inspired by the hierarchical structure and hunting dynamics of grey wolves^30^, employs a population division into four levels denoted as $\alpha ,\beta ,\delta$ and $\omega$. The uppermost level comprises the $\alpha$ wolf, followed by the $\beta$ wolf in the subsequent tier, and the $\delta$ wolf in the third tier. The remaining wolves, situated in the lowermost layer, are known as $\omega$ wolves or search wolves as seen in Fig. 1. The $\alpha ,\beta$ and $\delta$ wolves serve as leaders, each with a count of one. In GWO, the objective is for the $\omega$ wolves, representing the search wolves, to update their position and attain the optimal solution. Meanwhile, the $\alpha ,\beta ,$ and $\delta$ wolves represent the best, Second best, and third-best Solutions, respectively. The hunting behavior of grey wolves is primarily directed by the leading wolves ($\alpha ,\beta ,$ and $\delta$), guiding the iterative position updates of the search wolves ($\omega$) based on the leaders’ locations. This iterative process can be mathematically described as the formula governing the movement of the grey wolves in pursuit of their prey

1 $${\text{D}} = {\text{C}}*{\text{X}}_{p} \left( t \right) - {\text{X}}\left( t \right)$$

2 $${\text{X}}\left( {t + 1} \right) = {\text{X}}_{p} \left( t \right) - {\text{A}}*{\text{D}}$$

where $t$ represents the current iteration count, $*$ denotes the product operation, ${\text{X}}_{p}$ represents the position vector of the prey, ${\text{X}}$ represents the position vector of a grey wolf, and the calculation formulas for random vectors ${\text{A}}$ and ${\text{C}}$ are expressed as follows:

3 $${\text{A}} = 2{\text{a}}*{\text{r}}_{1} - {\text{a}}$$

4 $${\text{C}} = 2{\text{r}}_{2} .$$

**Figure 2:**
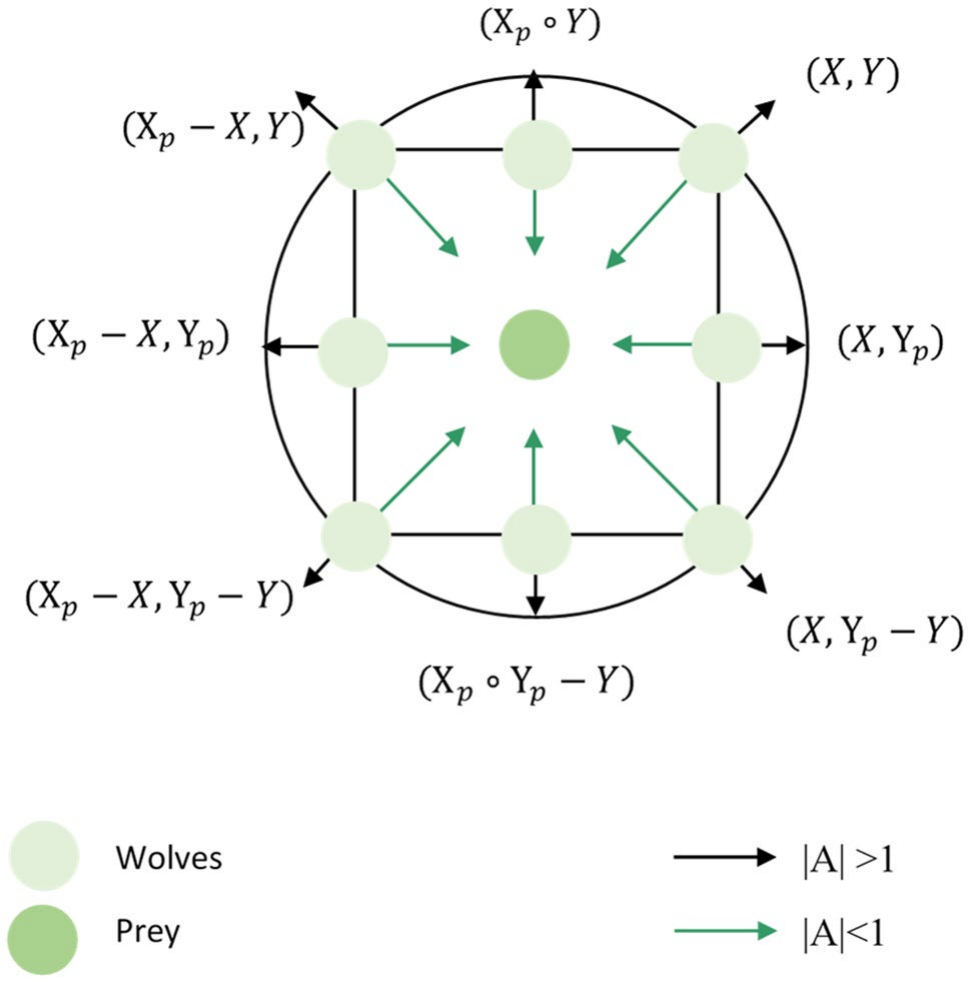
Illustration of search wolf during exploration and exploitation.

The utilization of random vectors and linearly decreasing values to optimize the position updates in GWO is discussed below. Figure 2 illustrates the potential areas that the $\omega$ wolf can occupy around the prey by adjusting the parameters ${\text{A}}$ and ${\text{C}}$. The random variables ${\text{r}}_{1}$ and ${\text{r}}_{2}$ aid the search wolves in reaching different points depicted in Fig. 2, both variables are within [0, 1], ${\text{a}}$ decrease from 2 to 0 over the increment of iteration number. The parameters ${\text{A}}$ and ${\text{C}}$ play a crucial role in the exploration and exploitation behaviour of GWO. ${\text{A}}$ takes on a random value within the range of [− ${\text{a}}$, ${\text{a}}$]. When ${\text{A}} > 1$ and ${\text{C}} > 1$, the population demonstrates a preference for exploration. Conversely, when ${\text{A}} < 1$ and ${\text{C}} < 1$, the population exhibits a tendency towards exploitation. The formulas governing the tracking of the grey wolves to target their prey are as follows:

5 $$\begin{array}{*{20}l} {\left\{ {\begin{array}{*{20}l} {{\text{D}}_{a} = {\text{C}}_{1} * {\text{X}}_{\alpha } - X} \hfill \\ {{\text{D}}_{\beta } = {\text{C}}_{2} *{\text{ X}}_{\beta } - X} \hfill \\ {{\text{D}}_{\delta } = {\text{C}}_{3} * {\text{X}}_{\delta } - X} \hfill \\ \end{array} } \right.} \hfill \\ {\left\{ {\begin{array}{*{20}l} {{\text{X}}_{1} = {\text{X}}_{\alpha } - {\text{A}}_{1} *{\text{D}}_{\alpha } } \hfill \\ {{\text{X}}_{2} = {\text{X}}_{\beta } - {\text{A}}_{2} * {\text{D}}_{\beta } } \hfill \\ {{\text{X}}_{3} = {\text{X}}_{\delta } - {\text{A}}_{3} *{\text{D}}_{\delta } } \hfill \\ \end{array} } \right.} \hfill \\ \end{array}$$

6 $${\text{X}}\left( {t + 1} \right) = \frac{{{\text{X}}_{1} + {\text{X}}_{2} + {\text{X}}_{3} }}{3}.$$

The distances between the lead wolves and search wolves in this situation are represented by the symbols ${\text{D}}_{a}$, ${\text{D}}_{\beta }$, and ${\text{D}}_{\delta }$, respectively. The locations of the lead wolves are shown by the symbols ${\text{X}}_{\alpha }$, ${\text{X}}_{\beta }$, and ${\text{X}}_{\delta }$. While ${\text{X}}_{1}$, ${\text{X}}_{2}$, and ${\text{X}}_{3}$ represent the step size and direction of the $\omega$ wolf towards the lead wolves, respectively, ${\text{C}}_{1}$, ${\text{C}}_{2}$, and ${\text{C}}_{3}$ are random vectors. Equation (6) is used to determine the wolf’s ultimate location. Algorithm 1 shows the iterative process of GWO.

**Figure:**
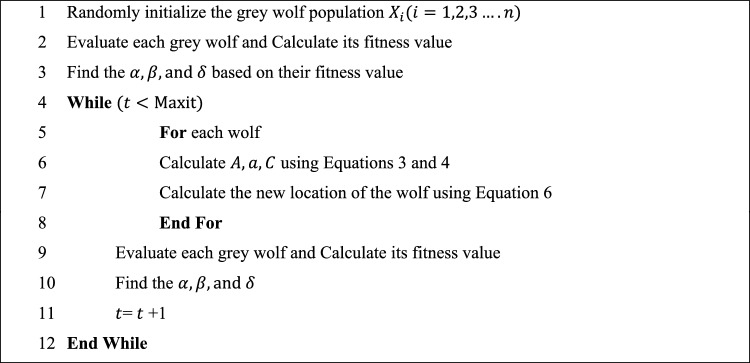
Algorithm 1 Steps of GWO

## Proposed CMWGWO

### Chaotic opposition learning (COL)

**Figure 3:**
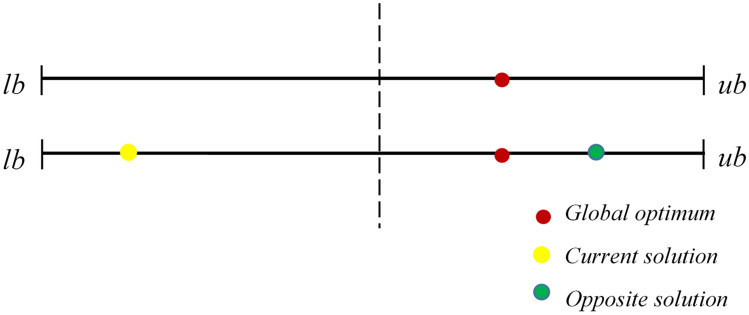
Graphical illustration of opposition learning.

Opposition-based learning (OBL) stands as a robust Optimizer improvement methodology in the domain of intelligence computation, initially introduced by Tizhoosh^51^. Generally, MAs begin with random initial solutions and iteratively strive to move closer to the global best solution. The termination of the search process occurs when specific predetermined requirements are met. In the absence of pertinent advance information about the solution, convergence might require a considerable amount of time. To address this, OBL incorporates a novel approach, depicted in Fig. 3, which involves assessing the fitness values of the current solution and the matching opposing solution at the same time. The superior individual is then retained for the next iteration, thereby promoting population diversity effectively. Notably, the opposite candidate solution has nearly a 50% higher chance of being closer to the global optimum compared to the current solution^52^. Consequently, OBL has gained widespread adoption as it significantly enhances the optimization performance of various MAs^53,54^. The mathematical representation of OBL is as follows:

7 $$\hat{X} = lb + ub - X.$$

The opposite solution is denoted by $\hat{X}$, while *X* represents the current solution. $lb$ and $ub$ correspond to the lower and upper limits of the search area. As evidenced by Eq. (7), OBL has the limitation of producing the opposite solution at a given position^55^. This approach proves effective during the initial optimization phases. However, as the search process advances, there is a possibility that the opposite solution may end up close to a local optimum. Consequently, other individuals in the population might rapidly gravitate towards this area, leading to premature convergence and reduced solution accuracy. In response to this issue, the random opposition-based learning (ROBL) strategy which incorporates random perturbations to modify Eq. (7) as follows was introduced in this work:

8 $$\hat{X} = lb + ub - rand*X$$

*rand* is an arbitrary value taken from the interval [0, 1]. While ROBL demonstrates some improvement in population diversity and is efficient in mitigating local optima, its convergence speed remains unsatisfactory. Chaos is the unpredictability observed in nonlinear structures, which possess dynamic, random, and ergodic properties. Incorporating chaos theory in algorithms facilitates the acceleration of convergence and strengthens the capability to maintain diversity. The CMWGWO includes a hybrid approach that combines normal OBL with chaotic maps, referred to as the chaotic opposition learning (COL) strategy. The mathematical expression for COL is provided below:

9 $$\widehat{{X^{CO} }} = lb + ub - \varphi *X$$

$\widehat{{X^{CO} }}$ represents the inverse solution of *X*, and φ denotes the value of the chaotic map. The chaotic Map introduced in this work is calculated as given in Eq. (10)

10 $$\varphi_{i + 1} = \left\{ {\begin{array}{*{20}l} {1,} \hfill & {\varphi_{i} = 0} \hfill \\ {\frac{1}{{mod\left( {\varphi_{i} ,1} \right)}},} \hfill & {\text{ otherwise }} \hfill \\ \end{array} } \right.$$

**Figure 4:**
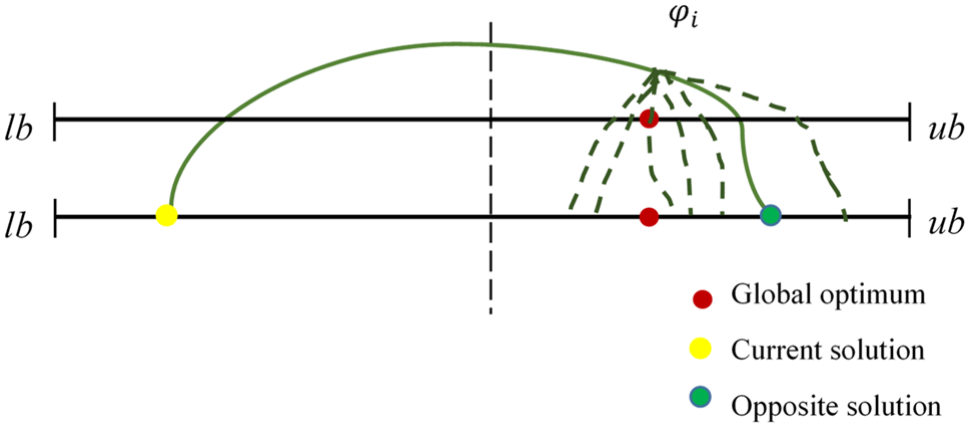
Graphical illustration of chaotic opposition learning.

The visual representation of the COL implemented in this work is displayed in Fig. 4. The illustration depicts that with the introduction of Chaos, the opposition solution, instead of falling in the same position can avoid getting trapped in the local Optimal by falling in random positions.

### Mirror reflection strategy (MRS)

**Figure 5:**
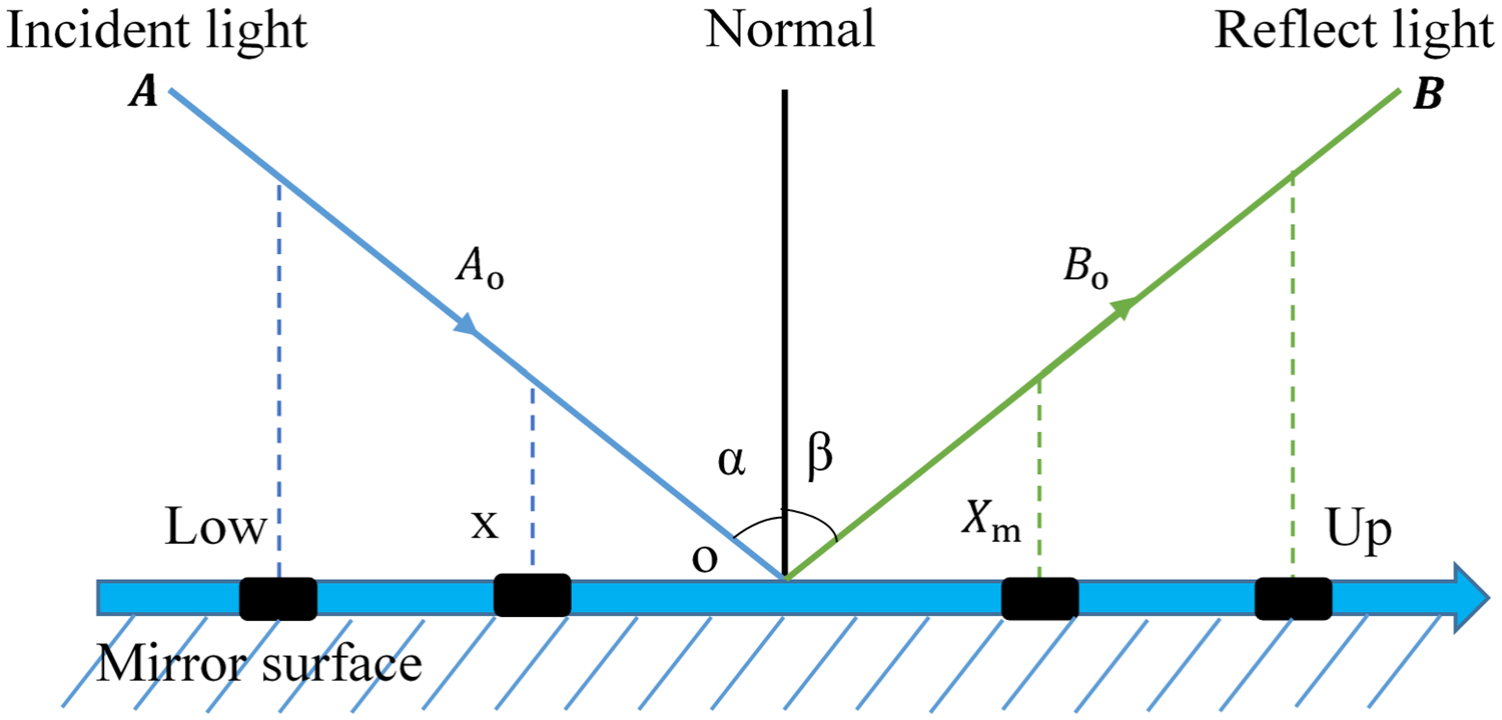
Illustration incident reflected light on a mirror surface.

The mirror reflection principle describes the phenomena that occur when light comes into contact with the boundary between two different media^56^. This principle comes into play when a portion of the incident light returns to the original medium. There are two basic rules that govern mirror reflection. Firstly, the angle at which the light is reflected (angle of reflection) is equivalent to the angle at which it strikes the surface (angle of incidence). Secondly, the reflected and the incident ray lie on opposite sides of an imaginary line denoted the "normal" that is perpendicular to the surface at the point of reflection. Drawing inspiration from these well-established principles, the proposed CMWGWO includes a Mirror Reflection Learning (MRL) strategy. In the MRL strategy, we represent the incident angle direction of a potential solution on the x-axis to denote its location. Simultaneously, the reflected angle direction on the x-axis represents the mirrored version of the solution. The MRL method explores both the potential solutions and their mirror reflections, to choose the best solution thereby expanding the search area. Figure 5 gives a visual demonstration of the concept of mirror-reflection learning. The potential answers are chosen within the $\left[ {lb,ub} \right]$ interval. The halfway between $lb$ and $ub$ is denoted by $O = \left( {X_{0} ,0} \right)$ and $X\left( {a,0} \right)$ denotes an arbitrary variable inside the same interval, $\left( {b,0} \right)$ is the location of $X_{m}$, the mirror reflection of $X$. The following Eqs. (11) to (14) define the relationship between incident and reflection angles and subsequently provide a method for determining the mirror-reflected solution. They are based on the first law of mirror reflection previously mentioned in the subsection: The angle of reflection is equal to the angle of incidence. Equation (11) and (12) establishes the relationship between the incident angle (α) and the reflection angle (β) using the tangent function:

11 $${\text{tan}}\alpha = \frac{{X_{0} - a}}{{A_{0} }}$$

12 $${\text{tan}}\beta = \frac{{b - X_{0} }}{{B_{0} }}.$$

By considering $\alpha$ as the incident angle and $\beta$ as the reflection angle, Eq. (13) can be derived following the first rule of reflection.

13 $$\frac{{X_{0} - a}}{{A_{0} }} = \frac{{b - X_{0} }}{{B_{0} }}$$

14 $${\text{b}} = \frac{{B_{0} \left( {X_{0} - a} \right)}}{{A_{0} }} + X_{0}$$

To simplify Eq. (14), a variable $\lambda_{m}$ is introduced such that $B_{0} = \lambda_{m} A_{0}$_,_ resulting in the following expression:

15 $$b = \left( {\lambda_{m} + 1} \right)X_{0} - \lambda_{m} a = \left( {0.5\lambda_{m} + 0.5} \right) \times \left( {lb{ } + ub} \right) - \lambda_{m} a.$$

Equation (16) provides the expression of $\uplambda _{{\text{m}}}$.

16 $$\lambda_{m} = \left\{ {\begin{array}{*{20}l} {1 + \mu Q,} \hfill & {{\text{if}}\;r_{1} > r_{2} } \hfill \\ {1 - \mu Q,} \hfill & {\text{otherwise }} \hfill \\ \end{array} } \right.$$

Here, µ and Q are the elasticity coefficients and neighborhood radius, both occurring inside the interval of [0,1], and *r*_1_ and *r*_2_ are arbitrary values between 0 and 1. The inverse solution's updated equation is expressed as follows:

17 $$X_{m} = \left( {0.5\lambda_{m} + 0.5} \right) \times \left( {{\text{ low }} + up} \right) - \lambda_{m} X*Levy().$$

In this work, we have uniquely incorporated the levy mechanism into Eq. (17). This incorporation is motivated by its potential to significantly contribute to the exploration–exploitation balance, which is a crucial aspect in improving the performance of CMWGWO. The Levy flight, inspired by the Levy distribution, possesses unique characteristics that facilitate long-range exploration in the search space^57^. By leveraging this feature, MRS can effectively escape local optima, thus promoting the exploration of promising regions that may lead to superior solutions. Moreover, the Levy flight mechanism enhances the algorithm's capability to diversify the search process, which helps maintain population diversity and mitigate premature convergence issues.

### Worst individual disturbance (WID)

**Figure 6:**
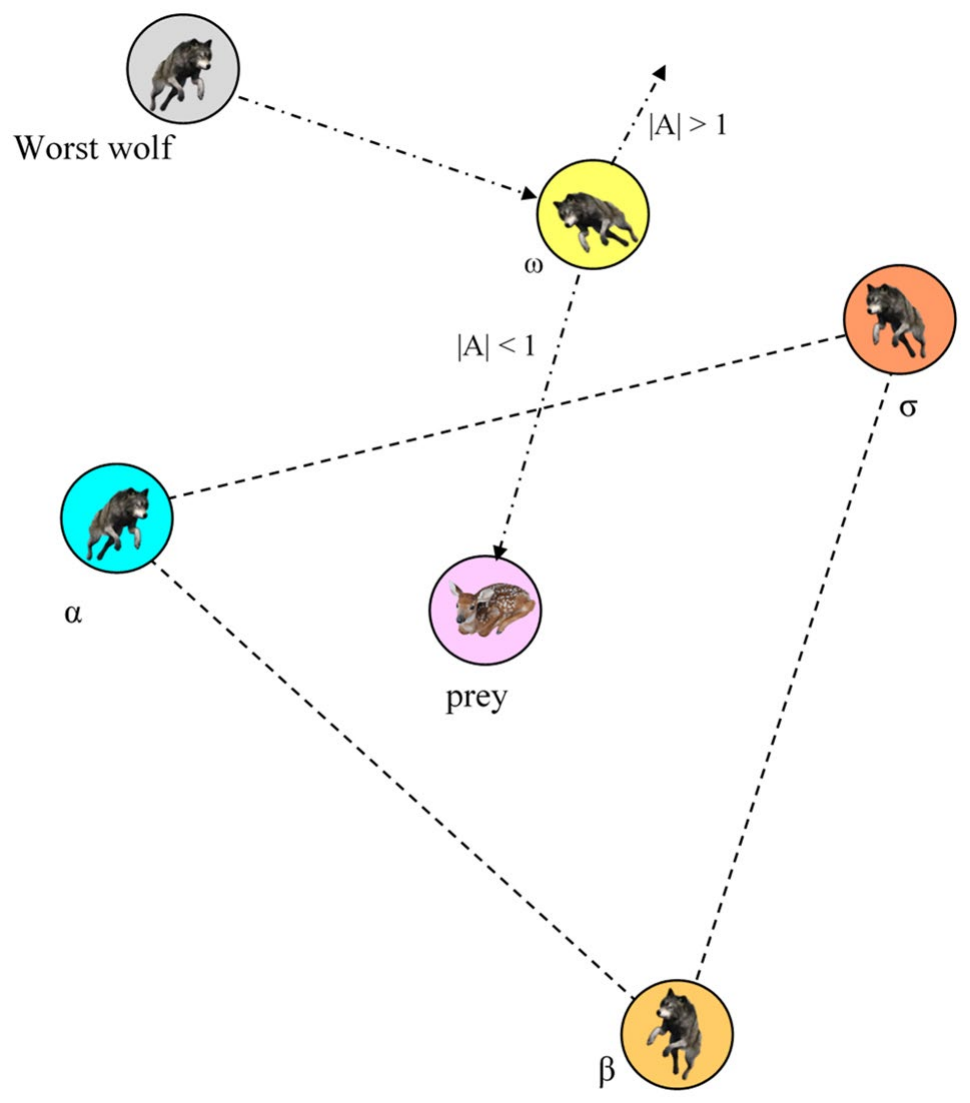
Information exchange between alpha wolf and worst wolf.

Majority of the improved variants of the GWO algorithm focus on increasing the chances of population individuals converging towards the best wolf. For example, the Grey Wolf Optimizer based on a new Weighted Distance (GWO-WD) introduced by Yan et al. eliminates and repositions several of the worst individuals^58^. However, it is important to reflect on the natural laws that Grey wolves must adhere to while hunting. During the process of surrounding their prey, Grey wolves encounter both the chance of successfully encircling the prey and the potential risk of the prey evading capture. This phenomenon is accurately modelled in the HHO algorithm that mimics the hunting behaviour of Harris hawks when they catch rabbits^59^. In HHO, there is a probability that the rabbit being chased by the hawk may escape. In that case, while the global optimal individual guides the entire population towards the best solution, there is a risk of getting stuck in a local optimum, leading to stagnation and failure to escape the local optimal space. Based on this idea, the proposed CMWGWO incorporates a worst individual disturbance strategy to escape local optima in case of unsuccessful encircling leading to a greater and more dynamic exploration of the search area as illustrated in Fig. 6, thus increasing the chances of finding better solutions. Equation (18) represents the encirclement phase, taking into account the global worst wolf:

18 $$X_{i}^{t + 1} = {\text{rand}}*X_{{\alpha { }}}^{t} - A*\left| {C*X_{{\alpha { }}}^{t} - X_{i}^{t} } \right| + \left( {1 - {\text{rand}}} \right)*X_{{\text{w }}}^{t} .$$

In the equation, $X_{{\text{w }}}^{t}$ represents the global worst wolf, and ${\text{rand}}$ is a randomly generated number from the interval [0,1]. ${\text{rand}}$ and (1–${\text{ rand}}$) are assigned randomly to $X_{{\alpha { }}}^{t}$ and $X_{{\text{w }}}^{t}$. Due to the uncertainty introduced by ${\text{rand}}$ and its random variation between 0 and 1, the search process is influenced not only by the global optimal individual but also by $X_{{\text{w}}}^{t}$. A higher value of ${\text{rand}}$ implies a more pronounced impact of the optimal individual on the formula, bringing the wolves closer to the target, effectively simulating a successful prey encirclement scenario. In contrast, if ${\text{rand}}$ is small, the impact of the worst individual on the formula becomes prominent, replicating the situation where wolves fail to encircle their prey effectively.

**Figure 7:**
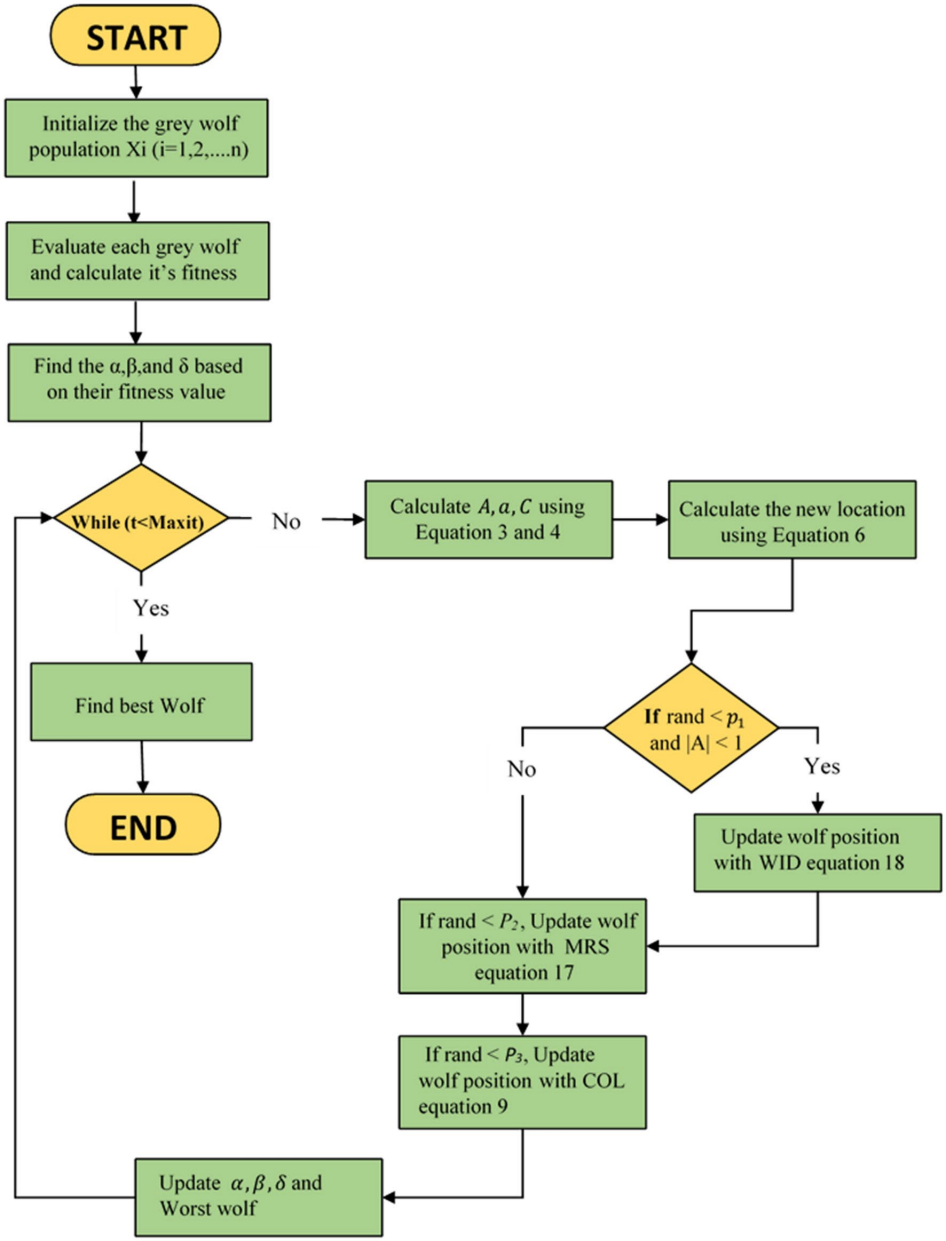
CMWGWO flow chart.

CMWGWO is an improved variant of the GWO algorithm, incorporating three novel techniques (WID, COL, and MRS) to enhance its performance. The algorithm starts by initializing a population of grey wolves as eventual solutions to an optimization problem. Each wolf's fitness is evaluated, and the best-performing wolves ($\alpha ,\beta ,{ }\delta$) and $Worst$ the Worst wolf are identified. The main loop iteratively updates wolf positions using calculated parameters $A,a,{\text{ and }}C$. The WID technique is applied with a probability of a random number less than $p_{1}$ and when |A|< 1 to some wolves. |A|< 1 implies the exploitation phase in other words, during this phase, if the best wolf gets trapped in suboptimal or the prey evades capture, the population can weaken the leadership of the best wolf to avoid convergence towards local optimal by using the information exchange between the best wolf and worst to break out of local optimal, furthermore the population is able to keep track of the prey effectively, followed by COL with a probability of $p_{3}$, and MRS with a probability of $p_{2}$ to improve diversity and amplify population exploration by expanding the search space respectively, all these improvements are subject to boundary constraints. The process continues until a termination condition is met. These newly introduced techniques aim to improve the exploration and exploitation abilities of the original GWO, potentially leading to improved optimization results. The step-by-step procedure of CMWGWO is expressed in Algorithm 2 and the graphical illustration is given in Fig. 7.

**Figure:**
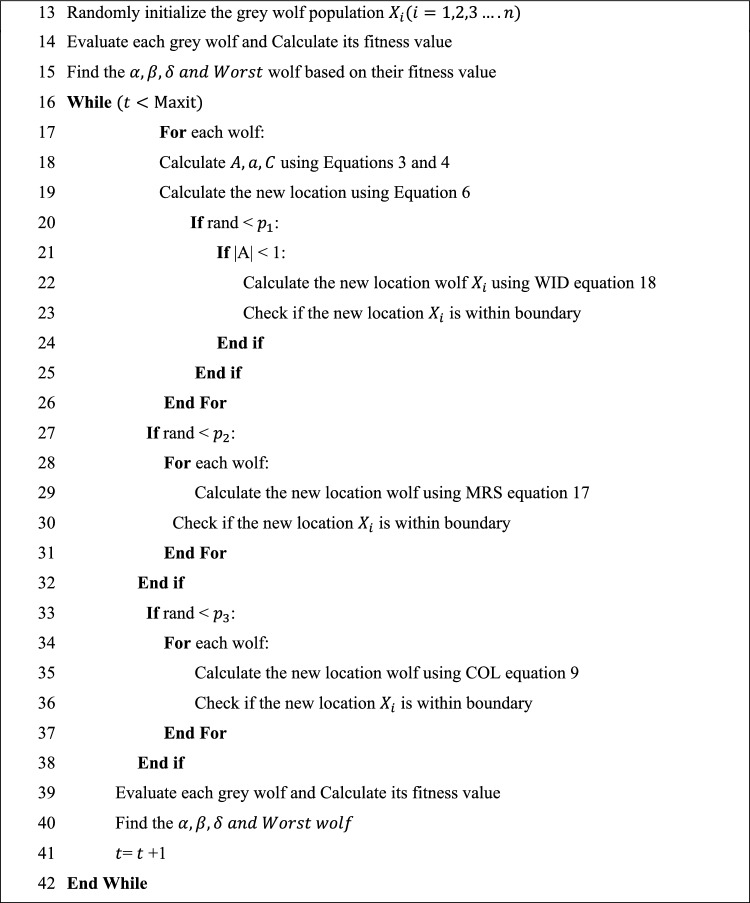
Algorithm 2 Steps of CMWGWO

## Computational complexity of CMWGWO

To analyze the computational complexity of the CMWGWO algorithm, we need to assess the complexity of each individual step and the number of iterations performed in the while loop. The breakdown of the steps and analysis of complexity is given below:

1. Random initialization: Initializing the grey wolf population $X_{i} \left( {i = 1,2,3 \ldots n} \right)$ involves generating random values for each individual wolf's position in the search space. The complexity of this step is $O\left( n \right)$, where $n$ is the size of the population and big $O$ denotes CMWGWO’s complexity^60,61^.
2. Fitness evaluation: Evaluating the fitness of each grey wolf requires evaluating the objective function of each individual. The computational complexity of this step depends on the complexity of the objective function and how it scales with the problem size. The complexity of evaluating the objective function as $O\left( {fitness} \right)$.
3. Finding the $\alpha ,\beta ,{ }\delta and Worst$: This step involves identifying the best, second-best, third-best, and worst grey wolves based on their fitness values. The complexity of finding these wolves is $O\left( n \right)$.
4. The main loop (While loop): The main optimization loop iterates until the termination condition is met $(t < {\text{Maxit}})$. The number of iterations is determined by ${\text{Maxit}}$, so we can denote the complexity of the while loop as $O\left( {{\text{Maxit}}} \right)$.
5. Calculations within the loop: Within each iteration of the while loop, there are three separate techniques included in traditional GWO. The complexity of each of these techniques can be denoted as $O\left( 1 \right)$ since they involve basic arithmetic operations and comparisons.
6. Boundary checks are carried out once each wolf's new position has been determined to make sure that it remains inside the bounds of the search area. The dimension of the search area and the effectiveness of the boundary-checking method determine how complicated these boundary checks are. Boundary check complexity is expressed as $O\left( {\text{d}} \right)$, where d is the search area 's dimensionality. The computational complexity of the CMWGWO algorithm can be approximated as expressed in Eq. (19), due to the introduction of these new techniques it is evident that the complexity of CMWGWO is higher than that of the original GWO:
  19 $$O\left( n \right) + O\left( {fitness} \right) + O\left( n \right) + O\left( {{\text{Maxit}}} \right) + {\text{Maxit}}*\left( {3*O\left( 1 \right) + n*\left( {O\left( 1 \right) + O\left( d \right)} \right)} \right).$$

## Experiments and result analysis

In this part, we will carry out tests to verify CMWGWO's efficacy while highlighting the improvement it offers. To confirm their effectiveness, each mechanism’s analysis will be used to comprehensively assess the improvement techniques used. To support the validity of CMWGWO's superiority, studies will also be undertaken to evaluate the optimization performance of CMWGWO with various improved versions of GWO. The enhanced GWO in this work will also be put up against original algorithms, further demonstrating the optimization value of GWO. Benchmarking the performance of several algorithms using a variety of complex tasks is an important step^62^. Therefore we will put CMWGWO through 23 benchmark functions, 10 CEC 2019 functions, and 3 real-world engineering situations to show its supremacy. The 23 benchmark functions are specifically described in Table 1, together with their mathematical formulations, dimensions, and theoretically ideal values. Researchers have carefully chosen these test functions from a list of frequently used CEC functions^63^. Table 1 displays a set of 7 unimodal functions (F1-F7), each containing a single minimum value. These functions are ideal for evaluating the algorithm's exploitation performance, as they test its ability to converge to the global minimum. Additionally, Table 1 includes 6 multimodal functions (F8-F13), which differ from F1-F7 by having numerous local optimal. These functions assess the algorithm's exploration capability^64^, as they require it to search for multiple optimal solutions. Their expressions are provided in Table 1. Moreover, F14-F23 are multimodal functions as well, but they have a fixed dimensionality. In addition to 23 functions, CEC 2019 functions (C1–C10) are employed. The intricacy of this test suite has been increased by shifting and rotating them relative to the usual functions. Table 2 includes the details of the test suite. Throughout this work we will carry out 500 iterations with a population size of 50, in order to preserve the validity of the studies 30 repeated runs will be carried out to lessen the effects of population randomness and population concentration brought on by randomness, and the average value (AVG), standard deviation (STD) and Best will indicate the outcomes of each algorithms optimization.

**Table 1:** Mathematical of 23 benchmark function.

Function	Range	Dim	Fmin
$f_{1} \left( x \right) = \sum_{i = 1}^{n} x_{i}^{2}$	[− 100,100]	30/100/200/500	0
$f_{2} \left( x \right) = \sum_{{f_{{{\text{min}}}} }}^{n} \left| {x_{i} } \right| + \prod_{i = 1}^{n} \left| {x_{i} } \right|$	[− 10,10]	30/100/200/500	0
$f_{3} \left( x \right) = \sum_{i = 1}^{n} \left( {\sum_{j - 1}^{i} x_{j} } \right)^{2}$	[− 100,100]	30/100/200/500	0
$f_{4} \left( x \right) = {\text{min}}\left\{ {\left| {x_{i} } \right|,1 \le i \le n} \right\}$	[− 100,100]	30/100/200/500	0
$f_{5} \left( x \right) = \sum_{i = 1}^{n - 1} 100^{i} \left( {x_{i + 1} - x_{i}^{2} } \right)^{2} + \left( {x_{i} - 1} \right)^{2}$	[− 30,30]	30/100/200/500	0
$f_{6} \left( x \right) = \sum_{i = 1}^{n} \left( {\left[ {x_{i} + 0.5} \right]} \right)^{2}$	[− 100,100]	30/100/200/500	0
$f_{7} \left( x \right) = \sum_{i = 1}^{n} ix_{i}^{4} + {\text{random}}\left[ {0,1} \right)$	[− 1.28,1.28]	30/100/200/500	0
$f_{8} \left( x \right) = \sum_{i = 1}^{n} - x_{i} {\text{sin}}\left( {\sqrt {\left| {x_{i} } \right|} } \right)$	[− 500,500]	30/100/200/500	− 418.9892 × dim
$f_{9} \left( x \right) = \sum_{i = 1}^{n} \left[ {x_{i}^{2} - 10{\text{cos}}\left( {2\pi x_{i} } \right) + 10} \right]$	[− 5.12,5.12]	30/100/200/500	0
$$\begin{aligned} f_{10} \left( x \right) & = - 20{\text{exp}}\left( { - 0.2\sqrt {\frac{1}{n}\sum_{i = 1}^{n} x_{i}^{2} } } \right) \\ & \quad - \;{\text{exp}}\left( {\left( {1/n} \right)\sum_{i = 1}^{n} {\text{cos}}\left( {2\pi x_{i} } \right)} \right) + 20 + e \\ \end{aligned}$$	[− 32,32]	30/100/200/500	0
$f_{11} \left( x \right) = 1/4000\sum_{i = 1}^{n} \sum x_{i}^{2} - \prod_{i = 1}^{n} {\text{cos}}\left( {x_{i} /\sqrt i } \right) + 1$	[− 600,600]	30/100/200/500	0
$$\begin{aligned} f_{12} \left( x \right) & = \pi /n\left\{ {\sum_{i = 1}^{n - 1} \left( {y_{i} - 1} \right)^{2} \left[ {1 + 10{\text{sin}}^{2} \left( {\pi y_{i + 1} } \right)} \right] + \left( {y_{n} - 1} \right)^{2} } \right\} \\ & \quad + \;\sum_{i = 1}^{n} u\left( {x_{i} ,10,100,4} \right) + \pi /n10{\text{sin}}\left( {\pi y_{1} } \right) \\ y_{i} & = 1 + x_{i} + \left( {1/4} \right)u\left( {x_{i} ,a,k,m} \right) = \left\{ {\begin{array}{*{20}l} {k\left( {x_{i} - a} \right)^{m} } \hfill & {x_{i} > a} \hfill \\ 0 \hfill & { - a < x_{i} < a} \hfill \\ {k\left( { - x_{i} - a} \right)^{m} } \hfill & {x_{i} < - a} \hfill \\ \end{array} } \right. \\ \end{aligned}$$	[− 50,50]	30/100/200/500	0
$$\begin{aligned} f_{13} \left( x \right) & = 0.1\left\{ {\sum_{i = 1}^{n} \left( {x_{i} - 1} \right)^{2} \left[ {1 + {\text{sin}}^{2} \left( {3\pi x_{i} + 1} \right)} \right] + \left( {x_{n} - 1} \right)^{2} \left[ {1 + {\text{sin}}^{2} \left( {2\pi x_{n} } \right)} \right]} \right\} \\ & \quad + \;0.1{\text{sin}}^{2} \left( {3\pi x_{1} } \right) + \sum_{i = 1}^{n} u\left( {x_{i} ,5,100,4} \right) \\ \end{aligned}$$	[− 50,50]	30/100/200/500	0
$f_{14} \left( x \right) = \left( {\left( {1/500} \right) + \sum_{j = 1}^{25} 1/j + \sum_{i = 1}^{2} \left( {x_{i} - a_{ij} } \right)^{6} } \right)^{ - 1}$	[− 65,65]	2	1
$f_{15} \left( x \right) = \sum_{i = 1}^{11} \left[ {a_{i} - x_{1} \left( {b_{i}^{2} + b_{i} x_{2} } \right)/b_{i}^{2} + b_{i} x_{3} + x_{4} } \right]^{2}$	[− 5,5]	4	003
$f_{16} \left( x \right) = 4x_{1}^{2} - 2.1x_{1}^{4} + 1/3x_{1}^{6} + x_{1} x_{2} - 4x_{2}^{2} + 4x_{2}^{4}$	[− 5,5]	2	− 1.0316
$f_{17} \left( x \right) = \left( {x_{2} - 5.1/4\pi^{2} x_{1}^{2} + 5/\pi x_{1} - 6} \right)^{2} + 10\left( {1 - \left( {1/8\pi } \right)} \right){\text{cos}}x_{1} + 10$	[− 5,5]	2	0.398
$$\begin{aligned} f_{18} \left( x \right) & = \left[ {1 + \left( {x_{1} + x_{2} + 1} \right)^{2} \left( {19 - 14x_{1} + 3x_{1}^{2} - 14x_{2} + 6x_{1} x_{2} + 3x_{2}^{2} } \right)} \right] \\ & \quad \times \;\left[ {30 + \left( {2x_{1} - 3x_{2} } \right)^{2} \times \left( {18 - 32x_{1} + 12x_{1}^{2} + 48x_{2} - 36x_{1} x_{2} + 27x_{2}^{2} } \right)} \right] \\ \end{aligned}$$	[− 2,2]	2	3
$f_{19} \left( x \right) = - \sum_{i = 1}^{4} c_{i} {\text{exp}}\left[ { - \sum_{j = 1}^{3} a_{ij} \left( {x_{j} - p_{ij} } \right)^{2} } \right]$	[1, 3]	3	− 3.86
$f_{20} \left( x \right) = - \sum_{i = 1}^{4} c_{i} {\text{exp}}\left[ { - \sum_{j = 1}^{j = 1} a_{ij} \left( {x_{i} - p_{ij} } \right)^{2} } \right]$	[0,1]	6	− 3.32
$f_{21} \left( x \right) = - \sum_{i = 1}^{5} \left[ {\left( {X - a_{i} } \right)\left( {X - a_{i} } \right)^{T} + c_{i} } \right]^{ - 1}$	[0,10]	4	− 10.1532
$f_{22} \left( x \right) = - \sum_{i = 1}^{F = 1} \left[ {\left( {X - a_{i} } \right)\left( {X - a_{i} } \right)^{T} + c_{i} } \right]^{ - 1}$	[0,10]	4	− 10.4028
$f_{23} \left( x \right) = - \sum_{i = 1}^{10} \left[ {\left( {X - a_{i} } \right)\left( {X - a_{i} } \right)^{T} + c_{i} } \right]^{ - 1}$	[0,10]	4	− 10.5363

**Table 2:** CEC 2019 test suite.

No	Function names	Range	Dim	$Fmin$
*c*_1_	Storn’s Chebyshev polynomial fitting problem	[$- 8,192,{ }8,192]$	9	1
*c*_2_	Inverse Hilbert matrix problem	[$- 16,384,{ }16,384]$	16	1
*c*_3_	Lennard–Jones minimum energy cluster	$\left[ { - 4,4} \right]$	18	1
*c*_4_	Rastrigin’s function	$\left[ { - 100,100} \right]$	10	1
*c*_5_	Griewank’s function	$\left[ { - 100,100} \right]$	10	1
*c*_6_	Weierstrass function	$\left[ { - 100,100} \right]$	10	1
*c*_7_	Modified Schwefel’s function	$\left[ { - 100,100} \right]$	10	1
*c*_8_	Expanded Schaffer’s F6 function	$\left[ { - 100,100} \right]$	10	1
*c*_9_	Happy Cat function	$\left[ { - 100,100} \right]$	10	1
*c*_10_	Ackley function	$\left[ { - 100,100} \right]$	10	1

### Statistical and non-parametric analysis of each improvement technique contribution

Three strategies WID, COL, and MRS, are used by the CMWGWO algorithm to improve optimization performance. Three GWO variations were evaluated on 23 functions to show the impact of various techniques on GWO. Each variant denotes the employment of a single strategy: WIDGWO denotes the only application of the WID strategy, COLGWO denotes the sole application of the COL strategy, and MRSGWO denotes the sole application of the MRS approach. CMWGWO stands for the entire combination of all three methodologies. By contrasting the AVG, STD, and Best of the outcomes attained by each method across several functions, as shown in Table 3, the impacts of these techniques on GWO's search capability can be investigated. The average and best values provided by the COLGWO, WIDGWO, and MRSGWO algorithms are typically better than those of the conventional GWO, demonstrating that these three optimization techniques significantly enhance the algorithm's optimization accuracy in both exploration and exploitation. Additionally, CMWGWO surpasses COLGWO, WIDGWO, and MRSGWO in the majority of functions when considering average values, best values, and standard deviations of their results, outperforming all three optimization procedures. This shows that using all three of these procedures together enhances GWO's optimization speed and guarantees stable optimization capability.

**Table 3:** Statistical and non-parametric test comparison of GWO outcomes using different techniques.

	CMWGWO	GWO	COLGWO	WIDGWO	MRSGWO
F1					
Avg	**4.64E−240**	1.73E−36	1.27E−76	1.26E−191	1.93E−53
Std	**0**	1.17E−36	2.43E−77	**0**	1.03E−53
Best	**6.03E−261**	1.44E−90	2.19E−194	6.94E−75	4.96E−38
F2					
Avg	**1.64E−128**	7.35E−22	3.37E−43	4.84E−104	1.02E−27
Std	**1.39E−128**	6.64E−22	2.86E−43	4.52E−104	6.32E−28
Best	**4.13E−136**	3.38E−50	1.42E−105	3.49E−33	1.83E−22
F3					
Avg	**1.22E−170**	4.85E−7	3.81E−50	2.82E−124	9.10E−11
Std	**0**	1.23E−7	3.14E−50	1.53E−124	5.37E−11
Best	**3.59E**−**188**	2.08E−63	4.24E−131	4.32E−25	1.02E−10
F4					
Avg	**2.97E**−**92**	7.45E−8	3.95E−30	8.63E−77	7.08E−11
Std	**2.04E**−**92**	7.14E−8	1.76E−30	7.14E−77	1.16E−11
Best	**1.00E**−**96**	6.77E−37	2.76E−79	9.79E−23	4.86E−9
F5					
Avg	1.18	2.66E+1	2.67E+1	2.76E+1	**1.31E**−**1**
Std	6.66E−1	7.27E−1	6.48E−1	6.06E−1	**9.57E**−**2**
Best	**3.82E**−**3**	2.60E+1	2.66E+1	3.62E−2	2.55E+1
F6					
Avg	**7.51E**−**6**	4.85E−1	3.51E−1	2.26	3.92E−5
Std	**5.81E**−**6**	2.83E−1	2.67E−1	3.04E−1	1.01E−5
Best	**3.33E**−**7**	2.08E−5	1.60	2.39E−5	3.81E−5
F7					
Avg	**4.76E**−**5**	1.50E−3	5.15E−5	1.02E−4	9.95E−4
Std	**3.85E**−**5**	6.22E−4	4.73E−5	9.38E−5	9.77E−4
Best	4.70E−6	**4.34E**−**7**	9.13E−6	3.30E−5	3.37E−4
F8					
Avg	− 1.06E+4	− 6.35E+3	− 5.73E+3	− 3.13E+3	− **1.09E**+**4**
Std	1.79E+3	1.00E+3	1.40E+3	**3.12E**+**2**	1.69E+3
Best	− 1.26E+4	− 8.06E+3	− 3.94E+3	− **1.26E**+**4**	− 8.06E+3
F9					
Avg	9.11	1.03E+1	**0**	**0**	1.53E+1
Std	6.47	1.00E+1	**0**	**0**	1.42E+1
Best	**0**	**0**	**0**	**0**	1.71E−13
F10					
Avg	**8.00E**−**16**	3.81E−14	4.00E−15	4.00E−15	5.07E−15
Std	7.98E−16	3.57E−15	**0**	**0**	4.98E−15
Best	**4.44E**−**16**	4.00E−15	4.00E−15	**4.44E**−**16**	3.24E−14
F11					
Avg	**0**	3.72E−3	**0**	**0**	1.70E−3
Std	**0**	2.50E−3	**0**	**0**	5.18E−4
Best	**0**	**0**	**0**	**0**	**0**
F12					
Avg	3.61E−5	2.48E−2	3.01E−2	1.52E−1	**4.15E**−**6**
Std	3.19E−5	1.14E−2	1.64E−2	3.49E−2	**1.88E**−**6**
Best	**4.83E**−**7**	6.53E−3	8.92E−2	1.36E−6	6.31E−3
F13					
Avg	4.77E−4	3.44E−1	3.96E−1	1.39	**5.44E**−**5**
Std	4.18E−4	1.56E−1	2.18E−1	1.93E−1	**2.65E**−**5**
Best	**4.67E**−**6**	2.97E−5	9.43E−1	1.94E−5	8.80E−2
F14					
Avg	**9.98E**−**1**	3.55	2.18	4.20	**9.98E**−**1**
Std	**0**	3.52	1.90	4.11	**0**
Best	**9.98E**−**1**	**9.98E**−**1**	**9.98E**−**1**	**9.98E**−**1**	**9.98E**−**1**
F15					
Avg	**3.13E**−**4**	2.38E−3	1.05E−3	9.80E−4	4.01E−4
Std	**1.30E**−**5**	5.16E−4	2.41E−5	4.05E−4	2.78E−4
Best	3.08E−4	**3.07E**−**4**	3.08E−4	**3.07E**−**04**	**3.07E**−**04**
F16					
Avg	− **1.03**	− **1.03**	− **1.03**	− **1.03**	− **1.03**
Std	**0**	**0**	**0**	**0**	**0**
Best	− **1.03E**+**00**	− **1.03**	− **1.03**	− **1.03**	− **1.03**
F17					
Avg	**3.98E−1**	**3.98E−1**	**3.98E−1**	**3.98E−1**	**3.98E−1**
Std	**0**	**0**	**0**	**0**	**0**
Best	3.98E−01	3.98E−01	3.98E−01	3.98E−01	**3.98E**−**01**
F18					
Avg	**3**	**3**	**3**	**3**	5.70
Std	**0**	**0**	**0**	**0**	1.04
Best	**3**	**3**	**3**	**3**	**3**
F19					
Avg	**− 3.86**	**− 3.86**	− 3.86	− 3.86	− 3.86
Std	**0**	1.83E−3	2.54E−3	3.79E−3	1.83E−3
Best	**− 3.86**	**− 3.86**	**− 3.86**	**− 3.86**	**− 3.86**
F20					
Avg	− 3.24	− 3.25	− 3.24	− 3.21	− **3.26**
Std	9.15E−2	7.68E−2	7.87E−2	9.98E−2	**6.75E−2**
Best	− **3.32**	− **3.32**	− **3.32**	− **3.32**	− **3.32**
F21					
Avg	− **9.99**	− 8.24	− 7.85	− 8.76	− 6.27
Std	**6.37E−1**	2.90	3.05	2.27	2.04
Best	− **1.02E**+**1**	− **1.02E**+**1**	− **1.02E**+**01**	− **1.02E**+**1**	− **1.02E**+**1**
F22					
Avg	− 1.03E+1	− **1.04E**+**1**	− 9.54	− 9.86	− 6.97
Std	5.65E−1	**0**	2.07	1.62	2.31
Best	− **1.04E**+**1**	− **1.04E**+**1**	− **1.04E**+**1**	− **1.04E**+**1**	− **1.04E**+**1**
F23					
Avg	− **1.05E**+**1**	− 1.03E+1	− 8.30	− 9.78	− 7.20
Std	**0**	9.73E−1	3.06	1.86	2.35
Best	− 1.05E+1	− 1.05E+1	− 1.05E+1	− 1.05E+1	− **1.05E**+**1**
*P* Value	–	2.54E−3	3.60E−3	2.47E−3	1.59E−1
(+|=|−)	–	(17|4|2)	(16|6|1)	(17|5|1)	(14|4|5)
FRD-AVG	1.80	3.63	3.17	3.24	3.15
RANK	1	5	3	4	2

Significant values are in [bold].

The nonparametric Wilcoxon signed-rank test was used across the 23 functions to compare the differences between the 4 distinct GWOs and CMWGWO in Table 3 at a significance threshold of 5% recorded as a *P* Value in Table 3. Table 3 also shows the contrast between CMWGWO and various GWOs. The symbols “+”, “−”, and “=” denote that CMWGWO is more superior to, less superior than, and identical to the comparison algorithm. According to the results, CMWGWO performs better than the original GWO in 17 out of 23 functions and is inferior to GWO in just 2 of them. Using the three strategies, CMWGWO exceeds COLGWO, CIGWO, and MRSGWO in 16 functions, 17 functions, and 14 functions, respectively. This indicates that the three strategies employed in CMWGWO complement each other, compensating for the shortcomings of GWO and significantly enhancing its performance across different test functions that test both the diversification and intensification capacity of CMWGWO. Notably, when comparing CMWGWO to other variants of GWO, including the traditional GWO in Table 3, the difference is less than 0.05 as indicated by the *P* Value, implying significant improvements in performance. The exception is MRSGWO, where CMWGWO shows no significant difference because it achieves similar results to MRSGWO in some functions, this also shows that the MRS being part of CMWGWO contributes to its exceptional performance. The Friedman Average (FRD-AVG) of CMWGWO is 1.80, ranking first among the five algorithms, and the FRD-AVG of the GWOs with other strategies is also smaller than that of the original GWO. This highlights that CMWGWO's overall performance surpasses other GWO variants and the traditional GWO in the comprehensive ranking using the Friedman Rank.

**Figure 8:**
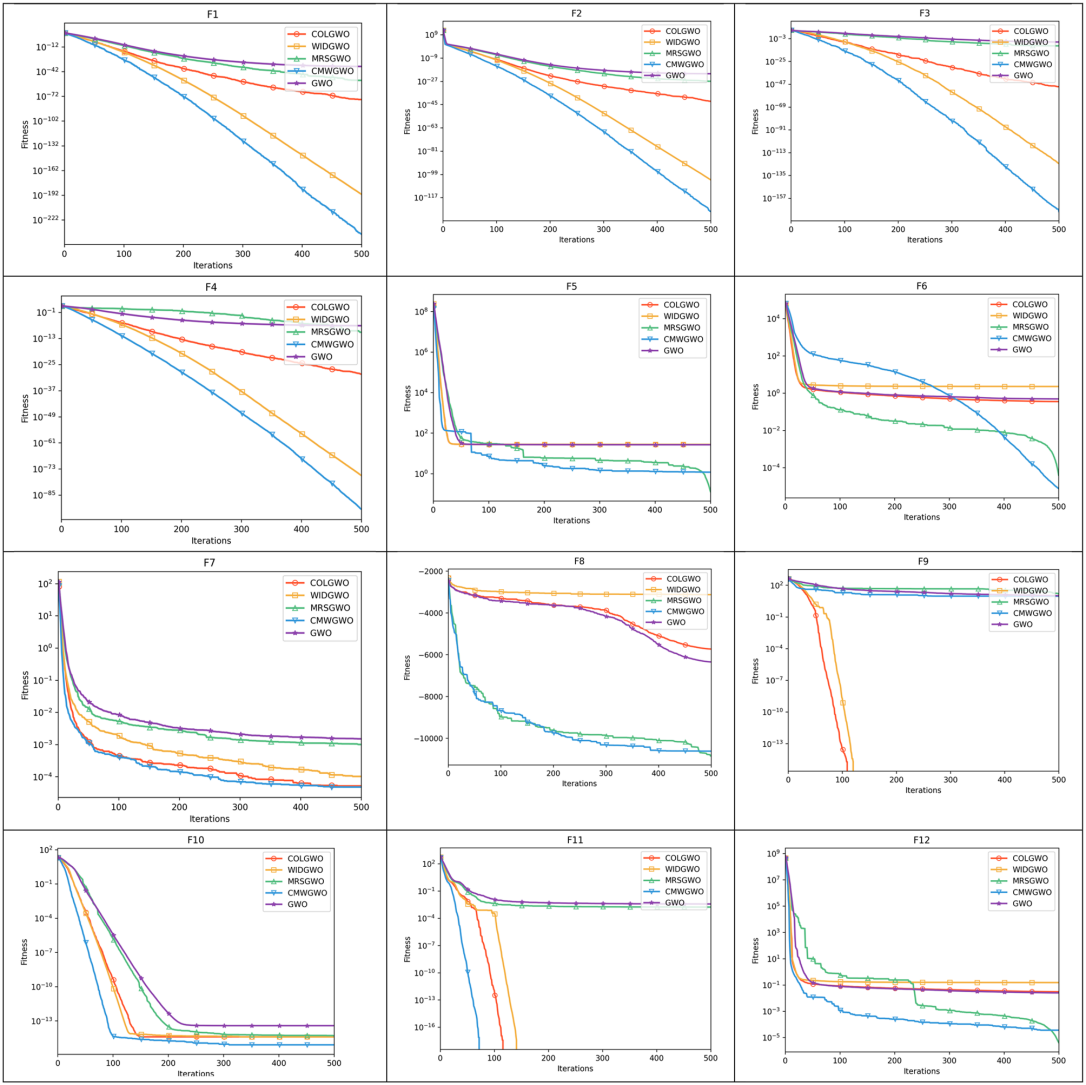

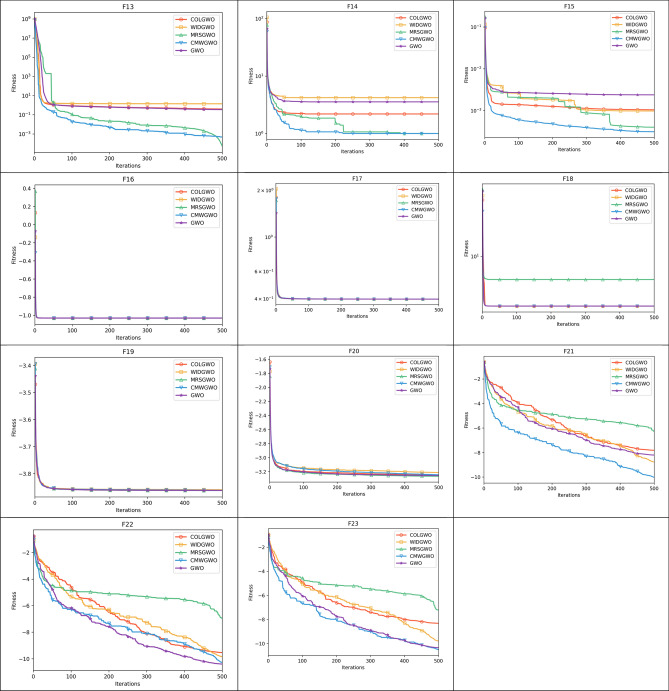
Convergence plot of different improvement techniques.

Figure 8 presents the convergence paths of CMWGWO and other variants based on different techniques, with the goal of evaluating the distinct performance of CMWGWO in achieving convergence while dealing with optimization functions. The study involves comparing CMWGWO with other algorithms derived from different techniques and the traditional GWO. The outcomes clearly demonstrate that CMWGWO outperforms the traditional GWO and other variants developed from different techniques in terms of convergence precision, particularly on all unimodal functions except F5. Remarkably, CMWGWO showcases exceptional convergence rates and successfully reaches the best optimal solution for F10, F11, F14-19, F21, and F23, showcasing its proficiency in handling multimodal functions. Comparatively, CMWGWO exhibits better convergence efficiency than GWO and other counterparts. These findings provide compelling evidence that the population diversification adjustments and the introduction of enhanced exploration techniques have significantly contributed to the success of CMWGWO. The experimental data strongly support the notion that CMWGWO has greatly improved its optimization capability and convergence performance.

### Dimension impact statistical analysis and non-parametric test of 23 test functions

CMWGWO is compared with several variants of GWO and Original algorithms, namely GWO^30^, Adaptive GWO (AdGWO)^39^, GWO based on Aquila exploration (AGWO)^65^, Augmented GWO & Cuckoo Search (AGWOCS)^66^, Random Walk GWO (RWGWO) ^41^, Hybrid-Flash Butterfly Optimization Algorithm (HFBOA) ^67^, Chimp Optimization Algorithm (CHOA)^68^, Particle Swarm Optimization (PSO)^69^ and Sine Cosine Algorithm ^70^ in this section on 23 functions while varying the dimension of each function. The parameters of this algorithm can be found in Table 4. Other parameters like iteration, population, and number of runs are set to 50, 500, and 30, respectively.

**Table 4:** Parameter settings.

Optimizer	Settings
AdGWO	$a_{0} = 2$, $\gamma = 0.95$
AGWO	$a_{0} = 2$, $B = 0.8$
AGWOCS	$a_{0} = 2$
RWGWO	$a_{0} = 2$
GWO	$a_{0} = 2$
HFBOA	$a = 0.1$, $p = 0.6$, $\mu = 4$, $\beta_{0} = 1,\alpha_{0} = 0.2$ and $c_{0} = 0.35$
CHOA	$f = \left[ {0,2.5} \right]$
PSO	$c_{1} ,c_{2} = 2,\omega_{2} = 0.9,\omega_{1} = 0.2$
SCA	$a = 2$
CMWGWO	$a_{0} = 2$, $p_{1} = 0.5 p2 = 0.1, p3 = 0.3$

By raising the dimension (Dim) of functions F1–F13 in the benchmark suite to 30, 100, 200, and 500, the effectiveness of CMWGWO in tackling high-dimensional optimization problems was assessed in this section. Tables 5, 6, 7 and 8 showcase the statistical experiment findings based on AVG (average), STD (standard deviation) and Best results for each function on the benchmark suite with Dim = 30, 100, 200, and 500, respectively, CMWGWO got remarkable FRD-AVG ranking values of 2.11, 1.73, 1.79, and 1.67, demonstrating that CMWGWO consistently ranks top across all dimensions it can be inferred that CMWGWO show robustness in complex problem handling as it maintain superior performance compared to other algorithm. The data shown in Tables 5, 6, 7 and 8 confirms the efficacy of each technique introduced to this variant of GWO.

**Table 5:** Statistical comparison of CMWGWO with GWO variants and original algorithms with Dim = 30.

	AdGWO	AGWO	AGWOCS	RWGWO	CHOA	HFBOA	PSO	SCA	GWO	CMWGWO
F1										
Avg	1.12E−59	1.31E−124	4.13E−51	5.89E−37	2.48E−8	9.97E−29	7.81E−6	3.87	1.73E−36	**4.64E**−**240**
Std	4.99E−60	9.63E−125	3.00E−51	5.72E−37	2.11E−8	9.70E−29	7.65E−6	2.28	1.17E−36	**0**
Best	8.34E−64	2.14E−127	3.36E−54	6.74E−39	4.03E−16	4.95E−63	5.91E−07	2.34E−02	4.96E−38	**6.03E**−**261**
F2										
Avg	1.73E−35	5.50E−55	1.20E−30	8.48E−22	2.62E−6	1.15E−1	6.34	7.65E−3	7.35E−22	**1.64E**−**128**
Std	1.64E−35	1.06E−55	9.66E−31	7.36E−22	1.62E−6	4.70E−2	5.43	5.81E−3	6.64E−22	**1.39E**−**128**
Best	3.30E−37	5.20E−57	2.38E−32	6.18E−23	4.31E−09	8.11E−15	9.46E−04	8.26E−05	1.83E−22	**4.13E**−**136**
F3										
Avg	1.61E−19	1.70E−5	6.16E−13	2.32E−6	2.64E−1	3.07E−21	4.17E+1	5.91E+3	4.85E−7	**1.22E**−**170**
Std	1.20E−19	2.30E−6	5.96E−13	1.26E−6	2.08E−1	1.82E−21	2.04E+1	5.82E+3	1.23E−7	**0**
Best	3.20E−28	9.28E−8	9.91E−18	1.21E−10	1.11E−08	1.24E−78	1.11E+01	3.44E+02	1.02E−10	**3.59E**−**188**
F4										
Avg	8.66E−14	7.59E−90	1.08E−13	6.47E−8	4.79E−3	1.68E−18	8.16E−1	2.45E+1	7.45E−8	**2.97E**−**92**
Std	6.62E−14	1.64E−90	1.07E−13	6.33E−8	1.09E−3	7.10E−19	2.48E−1	1.07E+1	7.14E−8	**2.04E**−**92**
Best	1.78E−19	1.00E−96	5.83E−16	8.06E−9	5.77E−6	4.35E−39	4.83E−1	5.25	4.86E−9	**1.00E**−**96**
F5										
Avg	2.85E+1	2.86E+1	2.67E+1	2.70E+1	2.90E+1	2.89E+1	7.90E+1	1.50E+4	2.66E+1	**1.18**
Std	3.49E−1	3.77E−1	5.83E−1	8.83E−1	6.79E−2	**3.20E**−**2**	5.39E+1	1.24E+4	7.27E−1	6.66E−1
Best	2.78E+1	2.75E+1	2.59E+1	2.58E+1	2.87E+1	2.88E+1	1.71E+1	2.99E+1	2.55E+1	**3.82E**−**3**
F6										
Avg	4.18	4.94	9.87E−1	2.90E−1	4.10	4.94	7.84E−4	9.61	4.85E−1	**7.51E**−**6**
Std	5.86E−1	5.58E−1	2.89E−1	2.34E−1	5.53E−1	3.39E−1	7.66E−4	6.67	2.83E−1	**5.81E**−**6**
Best	3.29	3.71	5.00E−1	2.90E−5	3.32	4.04	**9.31E**−**8**	4.25	3.81E−5	3.33E−7
F7										
Avg	4.99E−5	1.56E−3	1.09E−3	1.52E−3	8.82E−4	**2.81E**−**5**	2.95	5.60E−2	1.50E−3	4.76E−5
Std	4.53E−5	**6.28E**−**7**	7.00E−4	6.37E−4	7.44E−4	2.03E−5	1.79	5.36E−2	6.22E−4	3.85E−5
Best	1.11E−6	4.95E−6	1.91E−4	5.26E−4	1.49E−5	**1.03E**−**6**	3.82E−2	4.11E−3	3.37E−4	4.70E−6
F8										
Avg	− 2.76E+3	− 2.63E+3	− 8.65E+3	− 8.73E+3	− 5.75E+3	− 2.45E+3	− 5.66E+3	− 3.79E+3	− 6.35E+3	− **1.06E**+**4**
Std	3.91E+2	5.09E+2	7.72E+2	5.54E+2	**5.41E**+**1**	4.32E+2	1.25E+3	2.36E+2	1.00E+3	1.79E+3
Best	− 3.86E+3	− 3.84E+3	− 1.04E+4	− 9.94E+3	− 5.91E+3	− 3.39E+3	− 7.88E+3	− 4.28E+3	− 8.06E+3	− **1.26E**+**4**
F9										
Avg	**0**	**0**	3.79E−15	9.35	7.34E−2	**0**	9.71E+1	3.58E+1	1.03E+1	9.11
Std	**0**	**0**	3.39E−15	8.59	3.85E−2	**0**	3.42E+1	3.35E+1	1.00E+1	6.47
Best	**0**	**0**	**0**	5.68E−14	5.68E−14	**0**	3.71E+01	3.05E−02	1.71E−13	**0**
F10										
Avg	**4.44E**−**16**	**4.44E**−**16**	7.43E−15	3.89E−14	3.38E−5	**4.44E**−**16**	5.17E−3	1.40E+1	3.81E−14	8.00E−16
Std	**0**	**0**	6.48E−16	3.50E−15	1.92E−5	**0**	2.55E−3	8.46	3.57E−15	7.98E−16
Best	**4.44E**−**16**	**4.44E**−**16**	4.00E−15	2.89E−14	8.52E−9	**4.44E**−**16**	5.83E−4	2.42E−2	3.24E−14	**4.44E**−**16**
F11										
Avg	**0**	**0**	5.33E−4	1.53E−3	2.20E−3	**0**	8.20E−3	8.31E−1	3.72E−3	**0**
Std	**0**	**0**	4.95E−4	3.41E−4	1.88E−4	**0**	7.48E−3	4.32E−1	2.50E−3	**0**
Best	**0**	**0**	**0**	**0**	2.22E−16	**0**	8.09E−8	1.25E−01	**0**	**0**
F12										
Avg	6.31E−1	6.97E−1	8.28E−2	2.30E−2	5.97E−1	4.25E−1	6.93E−3	2.65E+1	2.48E−2	**3.61E**−**5**
Std	3.11E−1	2.01E−1	2.02E−2	1.37E−2	2.34E−1	1.21E−1	2.73E−3	1.43E+1	1.14E−2	**3.19E**−**5**
Best	3.24E−1	4.05E−1	5.62E−2	1.03E−5	3.61E−1	1.87E−1	**3.63E**−**9**	9.32E−1	6.31E−3	4.83E−7
F13										
Avg	2.32	2.61	9.23E−1	3.31E−1	2.84	2.49	3.67E−3	3.62E+3	3.44E−1	**4.77E**−**4**
Std	2.72E−1	1.67E−1	2.05E−1	1.61E−1	8.22E−2	2.82E−1	2.46E−3	3.52E+3	1.56E−1	**4.18E**−**4**
Best	1.69	2.08	4.58E−1	1.06E−1	2.58	1.45	**1.84E**−**7**	2.45	8.80E−2	4.67E−06
F14										
Avg	5.07	8.59	2.12	1.20	1.33	**9.98E**−**1**	1.98	1.53	3.55	**9.98E**−**1**
Std	3.20	3.84	1.91	5.46E−1	3.91E−1	**0**	1.85	8.91E−1	3.52	**0**
Best	2.01	2.21	**9.98E**−**1**	**9.98E**−**1**	**9.98E**−**1**	**9.98E**−**1**	**9.98E**−**1**	**9.98E**−**1**	**9.98E**−**1**	**9.98E**−**01**
F15										
Avg	1.95E−3	5.02E−3	3.84E−4	1.74E−3	2.12E−3	3.71E−4	4.47E−3	9.56E−4	2.38E−3	**3.13E**−**4**
Std	1.18E−3	4.21E−4	1.76E−4	4.30E−4	3.20E−4	4.37E−5	2.83E−3	3.76E−4	5.16E−4	**1.30E**−**5**
Best	5.75E−4	3.12E−4	3.09E−4	**3.07E**−**4**	1.51E−3	3.16E−4	3.64E−4	4.05E−4	**3.07E**−**4**	3.08E−4
F16										
Avg	**− 1.03**	**− **1.01	**− 1.03**	**− 1.03**	**− **1.02	**− **9.92E−1	**− 1.03**	**− 1.03**	**− 1.03**	**− 1.03**
Std	1.83E−3	4.64E−2	**0**	**0**	2.39E−2	6.53E−2	**0**	**0**	**0**	**0**
Best	**− 1.03**	**− 1.03**	**− 1.03**	**− 1.03**	**− 1.03**	**− 1.03**	**− 1.03**	**− 1.03**	**− 1.03**	**− 1.03**
F17										
Avg	4.42E−1	4.20E−1	**3.98E−1**	**3.98E−1**	1.18	**3.98E−1**	**3.98E−1**	3.99E−1	**3.98E**−**1**	**3.98E**−**1**
Std	7.55E−2	2.55E−2	**0**	**0**	6.54E−1	3.05E−4	**0**	8.61E−4	**0**	**0**
Best	**3.98E**−**1**	**3.98E**−**1**	**3.98E**−**1**	**3.98E**−**1**	4.19E−1	**3.98E**−**1**	**3.98E**−**1**	**3.98E**−**1**	**3.98E**−**1**	**3.98E**−**1**
F18										
Avg	3.17	3.04	**3.00**	**3.00**	3.06	3.01	**3.00**	**3.00**	**3.00**	**3.00**
Std	7.97E−1	1.26E−1	**0**	**0**	1.42E−1	8.60E−3	**0**	**0**	**0**	**0**
Best	**3**	**3**	**3**	**3**	**3**	**3**	**3**	**3**	**3**	**3**
F19										
Avg	− 3.78	− 3.80	− **3.86**	− **3.86**	− 3.47	− 3.43	− **3.86**	− 3.85	− **3.86**	− **3.86**
Std	1.57E−1	7.36E−2	2.54E−3	1.83E−3	2.71E−1	3.20E−1	**0**	4.50E−3	1.83E–3	**0**
Best	− **3.86**	− **3.86**	− **3.86**	− **3.86**	− 3.83	− 3.83	− **3.86**	− **3.86**	− **3.86**	− **3.86**
F20										
Avg	− 2.81	− 2.94	− **3.29**	− 3.25	− 1.79	− 1.95	− 3.24	− 2.97	− 3.25	− 3.24
Std	1.54E−1	2.24E−1	**3.97E−2**	6.91E−2	4.37E−1	4.05E−1	1.19E−1	2.40E−1	7.68E−2	9.15E−2
Best	− 3.07	− **3.22**	− **3.32**	− **3.32**	− 2.89	− 2.9	− **3.32**	− 3.2	− **3.32**	− **3.32**
F21										
Avg	− 2.69	− 2.80	− 6.21	− 9.18	− 7.40E− 1	− 5.22	− 7.24	− 3.14	− 8.24	− **9.99**
Std	1.59	1.41	1.98	2.08	**3.60E−1**	1.04	3.12	1.92	2.90	6.37E−1
Best	− 4.9	− 5.36	− 9.49	− **1.02E**+**1**	− 2.15	− 8.51	− **1.02E**+**1**	− 6.6	− **1.02E**+**1**	− **1.02E**+**1**
F22										
Avg	− 2.12	− 2.65	− 6.56	− 1.00E+1	− 8.66E−1	− 4.92	− 9.06	− 3.87	− **1.04E**+**1**	− 1.03E+1
Std	1.04	1.45	1.88	1.34	3.53E−1	6.08E−1	2.54	1.84	**0**	5.65E−1
Best	− 4.54	− 5.68	− 9.74	− **1.04E**+**1**	− 1.83	− 7.45	− **1.04E**+**1**	− 7.13	− **1.04E**+**1**	− **1.04E**+**1**
F23										
Avg	− 2.49	− 2.84	− 6.28	− 1.01E+1	− 1.02	− 5.22	− 9.81	− 4.23	− 1.03E+1	− **1.05E**+**1**
Std	1.32	1.53	2.24	1.74	3.39E−1	9.99E−1	2.13	1.36	9.73E−1	**0**
Best	− 5.57	− 7.51	− 9.99	− **1.05E**+**1**	− **2.29**	− 8.89	− **1.05E**+**1**	− 6.69	− **1.05E**+**1**	− **1.05E**+**1**
*P* Value	1.30E−3	8.76E−4	5.49E−3	5.39E−4	2.62E−4	3.59E−3	1.96E−4	5.96E−5	2.54E−3	–
(+|=|−)	(19|2|2)	(20|1|2)	(17|2|4)	(18|4|1)	(22|0|1)	(17|3|3)	(18|5|0)	(21|2|0)	(17|4|2)	–
AVG	5.98	6.57	4.22	4.24	7.7	5.57	6.15	7.8	4.67	2.11
RANK	6	8	2	3	9	5	7	10	4	1

Significant values are in [bold].

**Table 6:** Comparison of CMWGWO with GWO variants and original algorithms with Dim = 100.

	AdGWO	AGWO	AGWOCS	RWGWO	CHOA	HFBOA	PSO	SCA	GWO	CMWGWO
F1										
Avg	3.93E−23	8.27E−7	2.10E−21	1.60E−16	3.75E−3	1.96E−25	1.06E+1	9.34E+3	1.09E−16	**1.90E**−**183**
Std	7.74E−24	6.12E−7	1.80E−21	1.58E−16	1.95E−3	1.91E−25	2.85	5.27E+3	7.46E−17	**0**
Best	5.88E−31	1.37E−9	1.34E−22	2.52E−17	1.58E−6	3.87E−76	4.08	3.32E+3	2.50E−17	**1.18E**−**208**
F2										
Avg	9.99E−19	4.63E−8	2.72E−14	1.96E−10	4.95E−3	2.96E+48	1.11E+2	5.19	2.09E−10	**2.96E**−**100**
Std	7.74E−19	2.32E−9	1.85E−14	7.94E−11	3.81E−3	1.36E+48	2.91E+1	4.50	6.89E−11	**1.53E**−**100**
Best	1.80E−19	6.94E−10	1.06E−14	1.09E−10	8.99E−04	1.86E+34	5.15E+1	6.57E−1	1.08E−10	**1.85E**−**108**
F3										
Avg	2.44E−11	1.05E+3	5.68E+2	1.51E+3	8.19E+1	1.36E−19	1.30E+4	2.29E+5	8.62E+2	**2.43E**−**144**
Std	4.98E−12	1.68E+2	1.61E+2	1.49E+3	7.29E+1	2.73E−20	3.13E+3	4.38E+4	6.74E+2	**6.93E**−**145**
Best	2.45E−17	5.34E−5	6.80E−1	1.11E+2	2.49E−2	4.99E−67	7.90E+3	1.54E+5	5.38E+1	**7.18E**−**158**
F4										
Avg	2.26E−7	2.24E−47	2.62E−3	2.13E+1	3.71E−1	4.39E−20	1.01E+1	8.89E+1	9.95E−1	**3.89E**−**56**
Std	7.57E−8	1.68E−48	1.45E−3	1.58E+1	3.18E−1	3.21E−20	1.09	2.19	8.60E−1	**1.78E**−**56**
Best	1.70E−10	**1.04E**−**50**	5.36E−5	3.91E-1	1.75E−2	1.06E−49	7.17	8.44E+1	1.61E−1	5.48E−69
F5										
Avg	9.87E+1	9.88E+1	9.79E+1	9.72E+1	9.90E+1	9.89E+1	1.21E+4	1.12E+8	9.73E+1	**5.33E**+**1**
Std	2.61E−1	8.60E−2	6.36E−1	8.79E−1	1.46E−1	**4.07E**−**2**	1.15E+4	5.25E+7	7.74E−1	4.87E+1
Best	9.80E+1	9.86E+1	9.65E+1	9.58E+1	9.87E+1	9.89E+1	3.52E+3	3.55E+7	9.59E+1	**3.74E**−**3**
F6										
Avg	2.11E+1	2.17E+1	1.35E+1	7.44	1.93E+1	2.21E+1	1.03E+1	8.56E+3	7.70	**2.34E**−**3**
Std	1.28	6.97E−1	7.15E−1	1.05	8.39E−1	7.00E−1	4.48	4.99E+3	9.24E−1	**2.33E**−**3**
Best	1.90E+1	1.97E+1	1.16E+1	5.14	1.78E+1	1.97E+1	4.92	5.63E+2	6.03	**4.94E**−**6**
F7										
Avg	1.09E−4	6.27E−4	3.56E−3	5.28E−3	1.21E−2	**3.92E**−**5**	2.83E+2	1.33E+2	5.62E−3	5.14E−5
Std	8.18E−5	9.41E−5	1.91E−3	1.99E−3	8.02E−3	**3.37E**−**5**	1.32E+2	6.80E+1	2.44E−3	4.16E−5
Best	3.08E−6	2.24E−6	8.33E−4	2.35E−3	1.59E−4	**3.10E**−**7**	7.30E+1	3.17E+1	2.48E−3	5.09E−6
F8										
Avg	−5.02E+3	−4.40E+3	−2.77E+4	−2.34E+4	−1.82E+4	−4.58E+3	−1.30E+4	−6.96E+3	−1.63E+4	−**3.43E**+**4**
Std	7.15E+2	7.48E+2	1.71E+3	1.31E+3	**1.03E**+**2**	7.60E+2	3.76E+3	4.72E+2	4.26E+3	5.73E+3
Best	−6.89E+3	−5.83E+3	−3.18E+4	−2.67E+4	−1.84E+4	−6.40E+3	−1.99E+4	−7.95E+3	−2.00E+4	−**4.19E**+**4**
F9										
Avg	7.57E−15	**0**	1.86E−13	1.96E+1	1.28	**0**	7.32E+2	2.66E+2	1.93E+1	4.38E+1
Std	2.33E−15	**0**	9.18E−14	1.04E+1	6.88E−1	**0**	7.45E+1	1.27E+2	1.22E+1	4.03E+1
Best	**0**	**0**	**0**	1.25E−11	1.39E−4	**0**	5.58E+2	6.42E+1	1.24E−7	**0**
F10										
Avg	6.23E−14	7.81E−4	4.67E−12	1.26E−9	7.61E−3	**4.44E**−**16**	3.13	1.94E+1	1.31E−9	9.18E−16
Std	4.33E−14	5.22E−4	3.16E−12	5.19E−10	7.32E−3	**0**	2.64E−1	3.66	7.01E−10	8.17E−16
Best	7.55E−15	**4.44E**−**16**	8.03E−13	5.01E−10	4.12E−4	**4.44E**−**16**	2.27	5.59	4.49E−10	**4.44E**−**16**
F11										
Avg	3.70E−18	7.73E−2	3.70E−18	1.78E−3	2.60E−2	**0**	2.20E−1	9.89E+1	2.73E−3	**0**
Std	1.49E−18	3.94E−2	9.71E−19	4.52E−4	2.02E−2	**0**	4.27E−2	5.65E+1	4.04E−4	**0**
Best	**0**	**0**	**0**	2.22E−16	3.66E−5	**0**	1.37E−1	3.51	1.11E−15	**0**
F12										
Avg	9.90E−1	3.83E+4	4.05E−1	1.95E−1	8.79E−1	9.27E−1	2.62	2.72E+8	2.11E−1	**2.82E**−**5**
Std	8.05E−2	3.20E+4	5.74E−2	3.83E−2	9.56E−2	7.77E−2	1.40	1.09E+8	5.08E−2	**2.78E**−**5**
Best	8.30E−1	8.21E−1	3.21E−1	1.16E−1	7.03E−1	6.12E−1	7.94E−1	1.07E+8	1.48E−1	**1.06E**−**6**
F13										
Avg	9.64	9.75	7.60	5.98	9.81	9.82	3.46E+1	4.69E+8	5.90	**1.66E**−**3**
Std	1.61E−1	9.62E−2	3.83E−1	4.87E−1	1.16E−1	1.84E−1	1.56E+1	2.08E+8	3.98E−1	**1.65E**−**3**
Best	9.22	9.57	6.77	4.93	9.52	9.16	7.98	2.05E+8	4.99	**8.29E**−**5**
*P* Value	2.31E−2	1.59E−2	1.59E−2	1.59E−2	1.59E−2	1.82E−1	1.47E−3	1.47E−3	1.59E−2	–
(+|=|−)	(12|0|1)	(12|0|1)	(12|0|1)	(12|0|1)	(12|0|1)	(9|1|3)	(13|0|0)	(13|0|0)	(12|1|0)	–
FRD-AVG	4.27	4.82	4.64	6.45	6	3.18	8.45	9.82	5.64	1.73
RANK	3	5	4	8	7	2	9	10	6	1

Significant values are in [bold].

**Table 7:** Comparison of CMWGWO with GWO variants and original algorithms with Dim = 200.

	AdGWO	AGWO	AGWOCS	RWGWO	CHOA	HFBOA	PSO	SCA	GWO	CMWGWO
F1										
Avg	1.97E−13	8.51	5.36E−13	2.52E−10	5.66E−1	1.15E−24	2.24E+2	5.24E+4	2.47E−10	**3.02E**−**168**
Std	7.96E−14	3.27	4.45E−13	1.21E−10	5.10E−1	4.27E−25	3.05E+1	2.24E+4	9.98E−11	**0**
Best	1.89E−22	3.30E−02	8.96E−14	9.05E−11	1.42E−2	2.94E−81	1.47E+2	8.32E+3	1.08E−10	**1.77E**−**187**
F2										
Avg	2.80E−13	1.35E−4	2.99E−9	8.88E−7	1.06E−1	9.45E+100	4.16E+2	2.62E+1	9.12E−7	**4.05E**−**90**
Std	1.58E−13	1.21E−4	1.39E−9	2.46E−7	5.53E−2	6.43E+99	5.85E+1	1.88E+1	2.49E−7	**3.65E**−**90**
Best	8.22E−14	4.09E−6	1.21E−9	4.85E−7	3.28E−2	4.06E+88	3.16E+2	2.55	5.22E−7	**5.38E**−**98**
F3										
Avg	8.47E−8	4.19E+4	3.96E+4	4.54E+4	1.10E+3	6.17E−18	6.82E+4	9.42E+5	4.25E+4	**1.42E**−**136**
Std	1.58E−8	1.84E+4	3.35E+4	1.92E+4	6.56E+2	2.70E−18	1.57E+4	2.35E+5	1.66E+4	**1.28E**−**136**
Best	4.37E−12	5.86E−5	4.32E+2	1.80E+4	1.10E+1	9.60E−58	4.08E+4	5.79E+5	1.51E+4	**7.66E**−**151**
F4										
Avg	3.29E−6	**3.61E**−**74**	1.44E+1	3.76E+1	2.59	4.90E−19	1.75E+1	9.63E+1	2.45E+1	3.91E−38
Std	1.66E−6	**3.23E**−**74**	1.18E+1	1.34E+1	2.12	9.72E−20	1.25	7.36E−1	5.43	8.25E−39
Best	4.78E−9	**1.49E**−**76**	3.23E−1	1.74E+1	1.79E−1	3.14E−43	1.50E+1	9.40E+1	1.26E+1	6.35E−52
F5										
Avg	1.99E+2	2.00E+2	1.98E+2	1.98E+2	3.03E+2	1.99E+2	3.58E+5	5.53E+8	1.98E+2	**1.58E**+**2**
Std	1.83E−1	3.08	3.46E−1	6.40E−1	2.32E+2	**0**	5.27E+4	1.78E+8	6.29E−1	7.94E+1
Best	1.98E+2	1.99E+2	1.97E+2	1.96E+2	1.99E+2	1.99E+2	2.58E+5	2.70E+8	1.96E+2	**3.61E**−**1**
F6										
Avg	4.51E+1	6.12E+1	3.51E+1	2.45E+1	4.46E+1	4.74E+1	2.22E+2	4.90E+4	2.45E+1	**4.39E**−**3**
Std	1.40	4.91E+1	9.85E−1	1.45	3.05	5.76E−1	3.55E+1	2.30E+4	1.32	**4.38E**−**3**
Best	4.24E+1	4.53E+1	3.33E+1	2.07E+1	4.01E+1	4.53E+1	1.67E+2	9.57E+3	2.21E+1	**3.91E**−**4**
F7										
Avg	1.20E−4	2.52E−2	8.91E−3	1.23E−2	4.30E−2	7.94E−5	2.57E+3	1.47E+3	1.33E−2	**4.98E**−**5**
Std	1.15E−4	2.35E−3	4.44E−3	3.16E−3	2.33E−4	7.67E−5	5.03E+2	4.36E+2	4.07E−3	**3.65E**−**5**
Best	5.43E−6	2.49E−5	2.50E−3	8.07E−3	5.34E−4	**2.37E**−**6**	1.63E+3	3.78E+2	7.10E−3	7.91E−6
F8										
Avg	−6.96E+3	−6.28E+3	−5.68E+4	−3.92E+4	−3.59E+4	−6.28E+3	−1.99E+4	−1.04E+4	−2.48E+4	−**7.16E**+**4**
Std	8.06E+2	1.35E+3	2.48E+3	2.38E+3	**2.88E**+**2**	1.28E+3	6.75E+3	9.14E+2	1.06E+4	1.19E+4
Best	−8.52E+3	−9.95E+3	−6.27E+4	−4.32E+4	−3.63E+4	−8.69E+3	−3.45E+4	−1.25E+4	−3.64E+4	−**8.38E**+**4**
F9										
Avg	2.50E−13	1.25E+1	1.92E−9	4.32E+1	6.52	**0**	1.88E+3	5.37E+2	4.35E+1	1.08E+2
Std	5.54E−14	2.31	3.19E−10	2.05E+1	6.36	**0**	9.24E+1	1.73E+2	2.17E+1	1.03E+2
Best	**0**	**0**	9.09E−13	1.96E+1	3.25E−2	**0**	1.73E+3	1.82E+2	1.46E+1	**0**
F10										
Avg	1.77E−10	4.67E−5	5.28E−8	1.27E−6	4.46E−2	**4.44E**−**16**	5.91	1.96E+1	1.19E−6	1.04E−15
Std	8.02E−11	2.62E−5	2.72E−8	3.12E−7	3.33E−2	**0**	2.80E−1	3.40	3.09E−7	7.77E−16
Best	1.71E−12	**4.44E**−**16**	1.02E−8	8.46E−7	4.33E−3	**4.44E**−**16**	5.48	7.27	6.30E−7	**4.44E**−**16**
F11										
Avg	4.19E−15	5.40E−2	2.39E−3	3.21E−3	1.75E−1	**0**	1.10	4.59E+2	4.08E−3	**0**
Std	1.78E−15	4.31E−2	1.56E−4	1.96E−3	1.60E−1	**0**	2.76E−2	1.97E+2	2.81E−3	**0**
Best	**0**	**0**	1.90E−13	4.53E−11	4.13E−3	**0**	1.03	2.18E+2	2.22E−11	**0**
F12										
Avg	1.04	1.07	3.98E+1	4.41E−1	9.78E−1	1.05	2.64E+1	1.36E+9	4.30E−1	**2.15E**−**5**
Std	5.41E−2	5.16E−2	1.72E+1	5.93E−2	6.69E−2	4.38E−2	7.26	4.74E+8	4.59E−2	**1.80E**−**5**
Best	9.09E−1	9.93E−1	5.68E−1	3.49E−1	8.89E−1	9.36E−1	1.45E+1	6.70E+8	3.61E−1	**1.93E**−**8**
F13										
Avg	1.96E+1	3.59E+2	1.80E+1	1.63E+1	2.00E+1	1.99E+1	1.57E+3	2.52E+9	1.62E+1	**2.02E**−**3**
Std	1.03E−1	1.08E+2	4.74E−1	5.02E−1	4.04E−1	1.49E−1	9.22E+2	7.26E+8	5.72E−1	**1.91E**−**3**
Best	1.94E+1	1.96E+1	1.69E+1	1.55E+1	1.95E+1	1.94E+1	4.38E+2	1.35E+9	1.47E+1	**4.72E**−**5**
*P* Value	1.92E−2	1.59E−2	1.59E−2	1.59E−2	1.31E−2	9.12E−2	1.47E−3	1.47E−3	1.59E−2	–
(+|=|−)	(12|0|1)	(11|0|2)	(12|0|1)	(12|0|1)	(12|0|1)	(10|1|2)	(13|0|0)	(13|0|0)	(12|0|1)	**–**
FRD-AVG	4.21	6.71	4.33	4.96	6.00	3.88	8.67	9.58	4.88	1.79
RANK	3	8	4	6	7	2	9	10	5	1

Significant values are in [bold].

**Table 8:** Comparison of CMWGWO with GWO variants and original algorithms with Dim = 500.

	AdGWO	AGWO	AGWOCS	RWGWO	CHOA	HFBOA	PSO	SCA	GWO	CMWGWO
F1										
Avg	3.33E−8	2.19E+2	6.79E−4	4.96E−5	6.24E+1	9.53E−24	4.22E+3	1.81E+5	5.45E−5	**1.82E**−**149**
Std	3.16E−8	5.80E+1	6.64E−5	2.05E−5	5.59E+1	7.15E−24	3.05E+2	7.89E+4	1.80E−5	**1.40E**−**149**
Best	1.74E−15	8.29E−5	1.31E−5	2.14E−5	1.18E+1	6.98E−64	3.66E+3	7.62E+4	2.90E−5	**1.12E**−**169**
F2										
Avg	9.09E−9	1.53E−1	2.01E−5	1.31E−3	2.59	2.07E+265	1.12E+55	1.00E+2	1.38E−3	**1.50E**−**83**
Std	5.25E−9	2.32E−2	8.18E−6	1.44E−4	9.25E−1	4.14E+264	3.02E+54	6.27E+1	2.21E−4	**7.27E**−**84**
Best	2.96E−9	**0**	1.07E−5	1.03E−3	1.46	1.20E+253	1.18E+3	2.11E+1	9.05E−4	1.69E−89
F3										
Avg	2.96E−4	5.05E+5	4.71E+5	4.87E+5	4.04E+4	9.91E−18	4.85E+5	6.47E+6	4.63E+5	**1.60E**−**130**
Std	2.59E−4	3.54E+5	1.35E+5	1.05E+5	3.19E+4	4.30E−18	1.43E+5	1.30E+6	9.38E+4	**9.66E**−**131**
Best	1.65E−8	6.20E−6	1.56E+5	3.02E+5	6.23E+2	2.78E−59	3.19E+5	4.27E+6	2.49E+5	**5.11E**−**143**
F4										
Avg	9.34E−5	1.57E−1	4.03E+1	6.28E+1	8.15	4.93E−20	2.62E+1	9.89E+1	6.05E+1	**3.93E**−**25**
Std	1.04E−5	1.23E−1	3.21	4.08	5.59	7.29E−21	1.10	3.27E−1	3.87	**2.62E**−**25**
Best	8.72E−7	4.10E−5	3.47E+1	5.42E+1	1.83	2.62E−36	2.40E+1	9.81E+1	5.19E+1	**1.84E**−**37**
F5										
Avg	4.99E+2	3.72E+5	3.32E+6	4.97E+2	2.86E+3	4.99E+2	2.01E+7	1.95E+9	4.97E+2	**2.99E**+**2**
Std	**0**	1.37E+5	4.95E+5	3.65E−1	2.14E+3	**0**	2.09E+6	4.22E+8	2.63E−1	2.43E+2
Best	4.99E+2	4.99E+2	5.38E+2	4.96E+2	6.39E+2	4.99E+2	1.73E+7	1.05E+9	4.96E+2	**7.47E**−**2**
F6										
Avg	1.20E+2	2.01E+3	1.06E+2	8.53E+1	1.76E+2	1.22E+2	4.26E+3	1.95E+5	8.48E+1	**1.31E**−**2**
Std	1.55	1.07E+2	1.22	2.85	5.34E+1	7.30E−1	3.21E+2	7.23E+4	2.25	**9.79E**−**3**
Best	1.17E+2	1.22E+2	1.04E+2	7.97E+1	1.24E+2	1.21E+2	3.75E+3	3.05E+4	8.03E+1	**6.11E**−**4**
F7										
Avg	2.25E−4	1.00E+1	5.17E−1	4.30E−2	1.73E−1	8.90E−5	3.86E+4	1.38E+4	4.22E−2	**5.06E**−**5**
Std	8.62E−5	9.36	4.19E−1	1.10E−2	1.64E−1	8.12E−5	6.20E+3	3.11E+3	1.16E−2	**4.57E**−**5**
Best	4.69E−6	1.12E−5	5.65E−2	2.61E−2	2.35E−2	**1.97E**−**7**	2.56E+4	8.47E+3	2.62E−2	3.55E−6
F8										
Avg	− 1.09E+4	− 9.57E+3	− 1.43E+5	− 7.09E+4	− 8.89E+4	− 9.76E+3	− 3.37E+4	− 1.59E+4	− 5.69E+4	− **1.67E+5**
Std	1.69E+3	1.51E+3	2.86E+3	4.15E+3	**8.65E+2**	1.63E+3	1.17E+4	1.08E+3	2.00E+4	2.75E+4
Best	− 1.55E+4	− 1.21E+4	− 1.48E+5	− 8.05E+4	− 9.04E+4	− 1.30E+4	− 5.93E+4	− 1.94E+4	− 7.47E+4	− **2.09E+5**
F9										
Avg	2.22E−8	6.94E−1	6.15E−3	1.11E+2	7.57E+1	**0**	6.13E+3	1.08E+3	1.18E+2	1.18E+2
Std	1.12E−8	9.85E−2	2.62E−3	3.06E+1	4.42E+1	**0**	2.30E+2	4.76E+2	4.07E+1	4.81E+1
Best	1.82E−12	**0**	5.28E−7	5.70E+1	1.63E+01	**0**	5.75E+3	4.07E+2	5.92E+1	**0**
F10										
Avg	7.96E−6	1.87E−1	4.42E−4	3.35E−4	4.94E−1	**4.44E**−**16**	1.10E+1	2.00E+1	3.22E−4	1.75E−15
Std	3.95E−6	6.59E−2	2.99E−4	4.75E−5	2.68E−1	**0**	4.03E−1	2.47	6.55E−5	1.74E−15
Best	1.98E−9	4.00E−15	1.67E−4	2.64E−4	1.28E−1	**4.44E**−**16**	1.02E+1	1.19E+1	2.14E−4	**4.44E**−**16**
F11										
Avg	1.78E−12	3.07	2.00E−2	9.85E−3	1.39	**0**	2.26E+1	1.75E+3	6.51E−3	**0**
Std	1.05E−12	2.11	1.12E−2	2.21E−3	4.58E−1	**0**	7.76	6.67E+2	2.85E−3	**0**
Best	9.99E−16	**0**	8.43E−5	3.87E−6	7.66E−1	**0**	8.06	4.53E+2	2.98E−6	**0**
F12										
Avg	1.12	4.97E+5	4.25E+6	7.18E−1	6.32E+1	1.13	9.33E+4	5.89E+9	7.33E−1	**3.10E**−**5**
Std	2.54E−2	4.18E+5	2.19E+6	9.49E−2	4.36E+1	2.04E−2	3.57E+4	8.28E+8	7.32E−2	**2.79E**−**5**
Best	1.07	1.13	2.62E+5	5.94E−1	1.01	1.08	2.88E+4	3.86E+9	6.32E−1	**1.30E**−**6**
F13										
Avg	4.97E+1	2.33E+6	2.21E+7	5.15E+1	6.61E+4	5.00E+1	2.22E+6	9.77E+9	5.12E+1	**4.49E**−**3**
Std	1.24E−1	1.55E+6	2.00E+7	2.51	3.95E+4	9.32E−2	5.96E+5	2.21E+9	1.50	**4.22E**−**3**
Best	4.94E+1	4.97E+1	3.28E+3	4.81E+1	5.05E+1	4.97E+1	1.38E+6	4.93E+9	4.69E+1	**2.39E**−**5**
*P* Value	1.31E−2	5.77E−3	8.78E−3	7.13E−3	5.77E−3	6.19E−2	2.22E−3	1.47E−3	2.22E−3	–
(+|=|−)	(12|0|1)	(12|0|1)	(12|0|1)	(12|0|1)	(12|0|1)	(10|1|2)	(13|0|0)	(13|0|0)	(12|0|1)	**–**
FRD-AVG	3.63	7.42	6.08	4.88	5.83	3.25	8.17	9.58	4.50	1.67
RANK	3	8	7	5	6	2	9	10	4	1

Significant values are in [bold].

For functions F1–F7, in Table 5 (functions F1–F6), Table 6 (functions F1–F3, F5–F7), Table 7 (Functions F1–F3, F5–F7) and Table 8 (Functions F1–F7) CMWGWO obtained the best solution in these functions as the complexity of problem increased with the dimension. This suggests that CMWGWOO has the ability to converge to the global optimal value. This observation demonstrated that the CMWGWO has a high exploitative ability while solving unimodal functions compared to the original GWO. In addition, GWO variations such as AdGWO and AGWOCS produced competitive results. Moving on to F8-F13 in Tables 5, 6, 7 and 8, CMWGWO consistently outperforms other competitors and GWO variants, in functions F8, F11-F13. Furthermore, in Table 5, F21-F23, which are fixed-dimension functions, CMWGWO maintains superior performance in F14-F19, F21, and F23. The superior performance of CMWGWO can be attributed to the improvement strategies. COL maintains high diversity during optimization, MRS improves population exploration capacity, and WID enhances the population's ability to approach the optimal solution while reducing the dominance of the best wolf in order to escape local optima in multi-peaked problems (F13-F24). The *P* Value results from the Wilcoxon signed-rank test on the 23 benchmark suite at Dim = 30, 100, 200, and 500, shown in Tables 5, 6, 7 and 8, confirm that CMWGWO is significantly superior to other competitors. The statistical analysis further verifies that CMWGWO effectively enhances optimization performance in the search process.

### Statistical and non-parametric analysis of CEC 2019 functions

To evaluate the proposed optimizers performance in intricate objective functions, the AVG, STD, and Best were used as assessment metrics to gauge the precision as well as the reliability of the CMWGWO and other optimizers. It is evident from the statistics in Table 9 that CMWGWO obtains the most optimum solution for five out of ten functions. It is extremely crucial to highlight that the effectiveness of CMWGWO is a substantial advancement beyond the traditional GWO as well as other methods in C1, C4, C6, C7, C8, and C9 in terms of AVG. This significant enhancement is anticipated as a result of the addition of improvement strategies to enhance CMWGWO’s capacity to enhance local and global search while preserving variety. As a consequence, CMWGWO’s overall performance has significantly improved. Based on the Wilcoxon signed-rank test the *P* Values in Table 9, CMWGWO shows statistically significant improvement compared to AdGWO, AGWO, CHOA, HFBOA, GWO, AGWOCS, RWGWO, PSO, and SCA (*P* < 0.05). The Friedman test ranks CMWGWO as the best-performing algorithm among the ten, indicating its overall superiority in terms of these metrics. This shows that with MRS, COL, and WID, CMWGWO is able to maintain stability in overcoming local optimal and keeping population diversity consistent throughout the iteration process in challenging problems.

**Table 9:** Statistical comparison of CMWGWO with GWO variants and original algorithms on CEC 2019.

	AdGWO	AGWO	AGWOCS	RWGWO	CHOA	HFBOA	PSO	SCA	GWO	CWGWO
C1										
Avg	1	**1**	4.754E+6	**1**	1.006	**1**	8.743E+8	4.679E+6	7.536E+4	7.426E+4
Std	1.449E−10	**0**	4.496E+6	7.418E−8	3.112E−2	**0**	5.207E+8	4.248E+6	4.128E+4	3.386E+3
Best	**1**	**1**	1.009	**1**	**1**	**1**	1.606E+8	4.301E+3	2.027E+1	**1**
C2										
Avg	4.814	8.670E+1	6.756E+3	**4.334**	7.811	5	3.189E+4	4.729E+3	6.632E+2	9.071E+2
Std	3.140E−1	4.623E+1	1.834E+3	1.897E−1	6.584	**2.464E**−**3**	8.466E+3	2.137E+3	3.893E+2	5.668E+2
Best	4.267	5	3.608E+3	**4.218**	4.655	4.986	1.731E+4	1.782E+3	1.644E+2	7.330E+1
C3										
Avg	9.299	9.168	4.340	4.438	9.400	5.214	7.565	9.209	**4.216**	4.330
Std	7.188E−1	1.111	1.067	1.628	6.000E−1	**2.521E**−**1**	1.464	1.425	2.852	2.648
Best	7.394	6.563	2.597	**1.004**	8.188	4.423	4.609	6.628	1.411	1.413
C4										
Avg	7.614E+1	8.533E+1	3.748E+1	3.885E+1	1.206E+2	8.221E+1	4.040E+1	5.172E+1	2.143E+1	**1.575E+1**
Std	1.319E+1	1.117E+1	8.041	7.709	9.305	1.265E+1	1.246E+1	7.286	1.271E+1	**4.324**
Best	4.676E+1	6.771E+1	2.130E+1	2.674E+1	1.010E+2	5.994E+1	1.891E+1	3.952E+1	**5.778**	7.008
C5										
Avg	3.105E+1	4.062E+1	3.432	3.531	1.420E+2	5.796E+1	**1.156**	1.214E+1	2.211	1.399
Std	1.630E+1	1.712E+1	9.553E−1	2.038	2.786E+1	1.904E+1	**9.247E**−**2**	3.661	2.030	1.496E−1
Best	1.333E+1	9.521	2.116	2.144	8.929E+1	2.880E+1	**1.039**	6.682	1.242	1.169
C6										
Avg	9.894	9.694	4.595	5.357	1.136E+1	1.218E+1	3.811	7.877	2.744	**2.552**
Std	1.344	1.709	**8.233E**−**1**	1.035	1.230	1.057	1.605	1.313	1.032	1.204
Best	7.377	6.894	2.948	3.980	9.011	8.786	**1.002**	5.940	1.355	1.192
C7										
Avg	2.121E+3	2.367E+3	1.283E+3	1.392	1.999E+3	1.725E+3	1.145E+3	1.597E+3	9.047E+2	**7.220E+2**
Std	2.386E+2	2.868E+2	2.953E+2	2.678	1.684E+2	1.878E+2	2.938E+2	**1.646E+2**	4.690E+2	3.016E+2
Best	1.548E+3	1.662E+3	8.144E+2	9.256	1.512E+3	1.139E+3	5.975E+2	1.206E+3	**1.325E+2**	1.810E+2
C8										
Avg	5.046	5.197	3.997	4.220	5.199	5.186	4.287	4.572	4.021	**3.653**
Std	2.152E−1	2.390E−1	3.652E−1	4.092E−1	**1.131E**−**1**	2.090E−1	4.297E−1	2.007E−1	4.610E−1	5.217E−1
Best	4.358	4.367	3.198	3.092	4.960	4.724	3.271	3.977	2.817	**2.704**
C9										
Avg	2.245	2.427	1.340	1.352	3.267	4.441	1.236	1.639	1.223	**1.219**
Std	4.894E−1	7.474E−1	7.861E−2	8.106E−2	7.781E−1	4.956E−1	1.245E−1	1.557E−1	7.844E−2	**5.521E**−**2**
Best	1.427	1.425	1.225	1.212	1.839	3.241	**1.079**	1.396	1.126	1.117
C10										
Avg	2.151E+1	2.153E+1	2.102E+1	**2.037E+1**	2.149E+1	2.131E+1	2.132E+1	2.129E+1	2.147E+1	2.057E+1
Std	1.765E−1	1.041E−1	1.833	2.595	**7.569E**−**2**	8.690E−2	1.655E−1	9.469E−1	1.186E−1	3.655
Best	2.115E+1	2.124E+1	1.248	1.302E+1	2.132E+1	2.112E+1	2.102E+1	1.632E+1	2.119E+1	**1.232E**
*P* Value	3.329E−3	3.329E−3	5.062E−3	5.751E−3	3.329E−3	3.329E−3	9.344E−3	5.062E−3	1.097E−2	–
(+|=|−)	(8|0|2)	(8|0|2)	(10|0|0)	(7|0|3)	(8|0|2)	(8|0|2)	(9|0|1)	(10|0|0)	(7|1|2)	–
FRD-AVG	6.70	7.45	4.50	3.65	8.30	6.75	5.10	6.50	3.60	2.45
RANK	7	9	4	3	10	8	5	6	2	1

Significant values are in [bold].

### Convergence and box plot analysis on 23 functions and CEC 2019 functions

**Figure 9:**
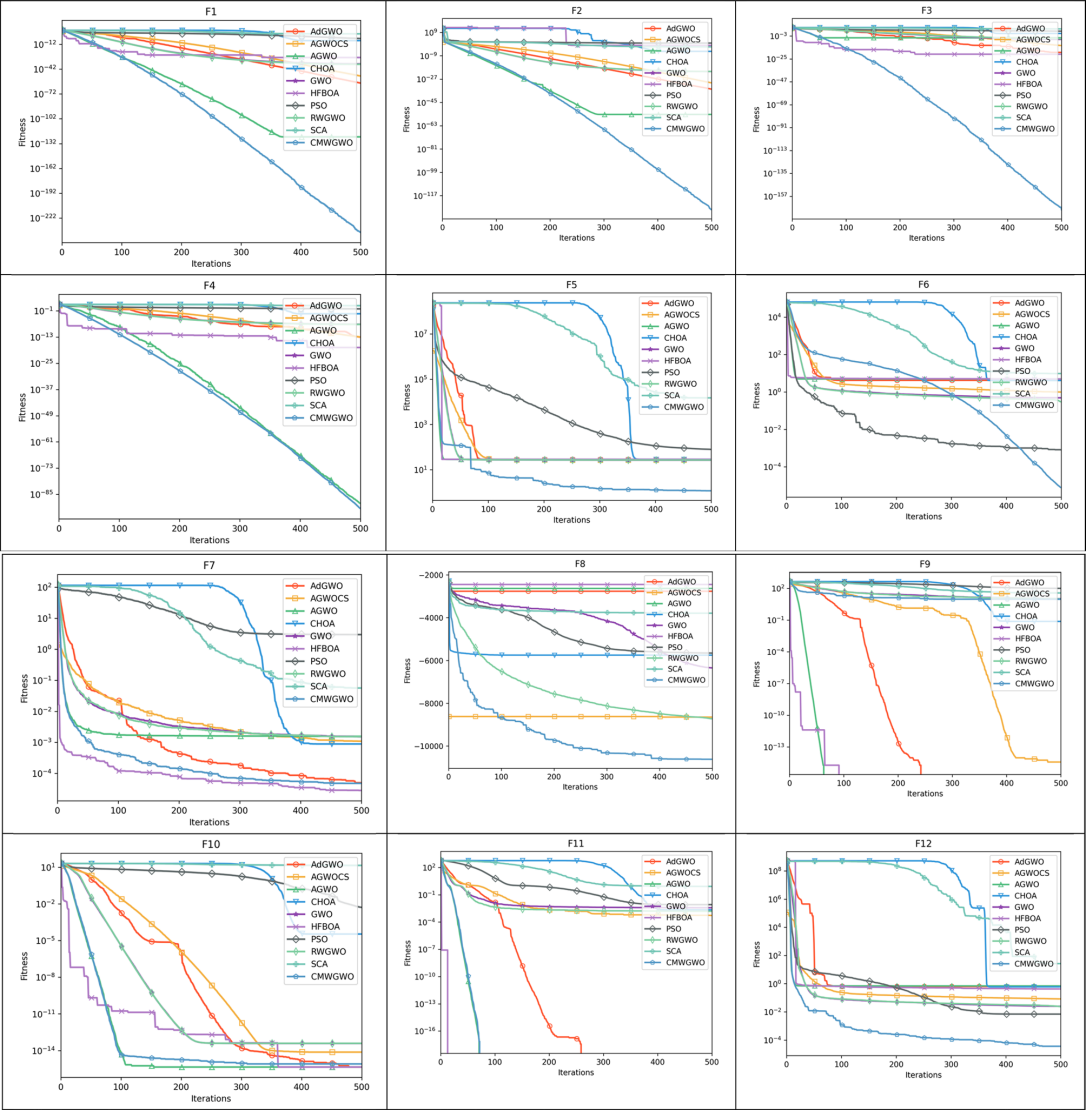

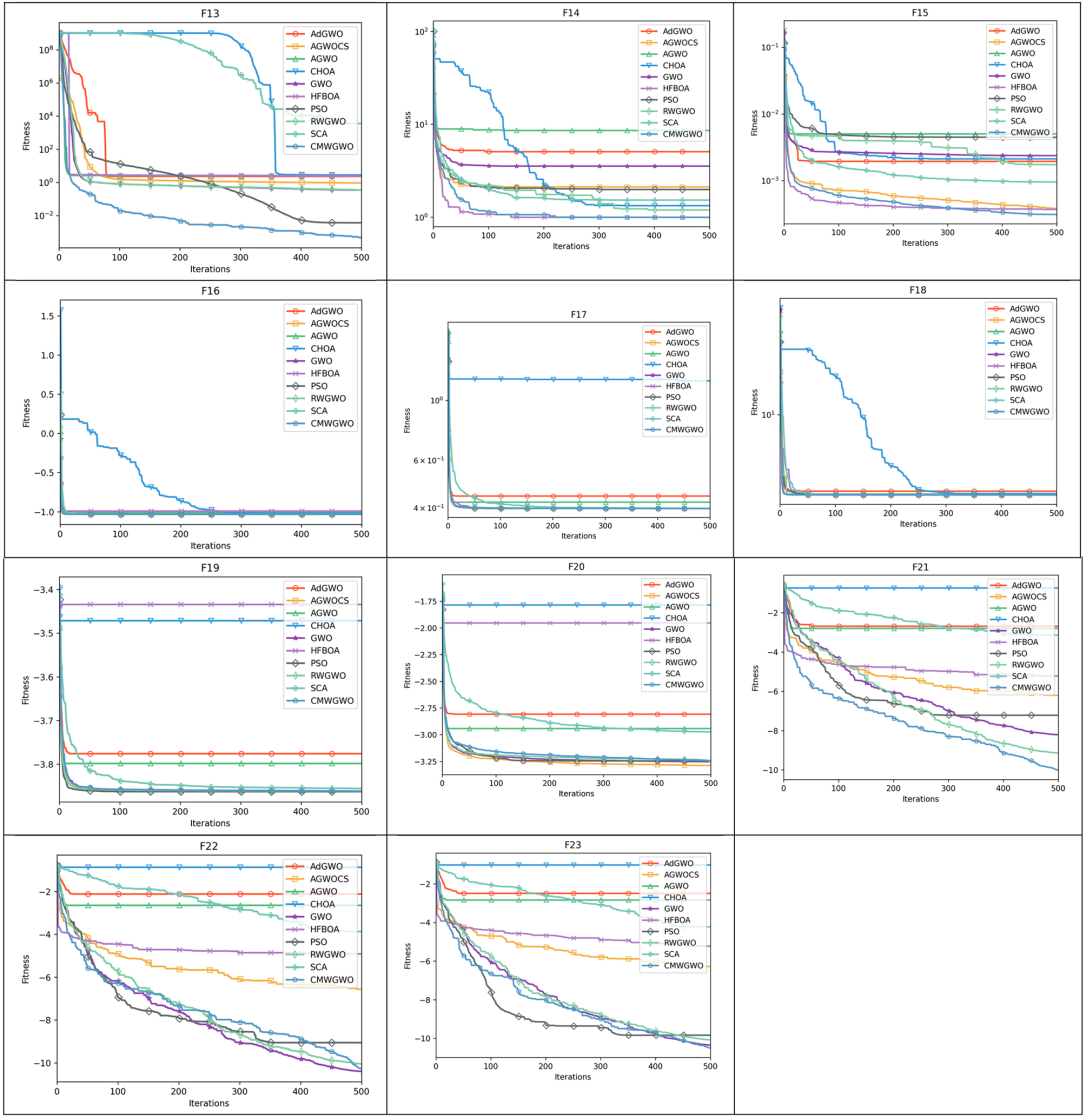
Convergence trajectory of CMWGWO and nine compared optimizers on 23 functions.

**Figure 10:**
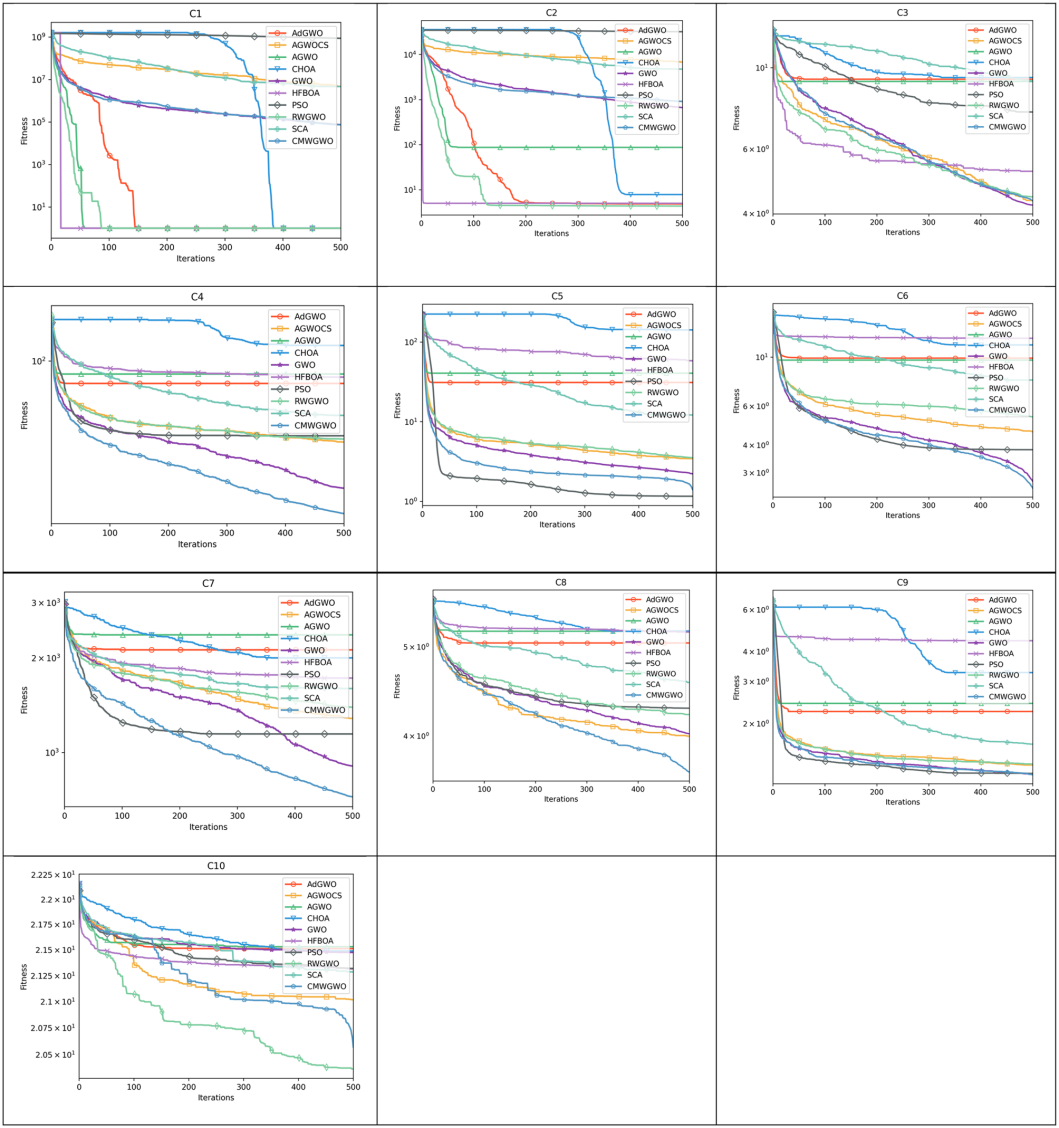
Convergence trajectory of CMWGWO and nine compared optimizers on CEC 2019.

**Figure 11:**
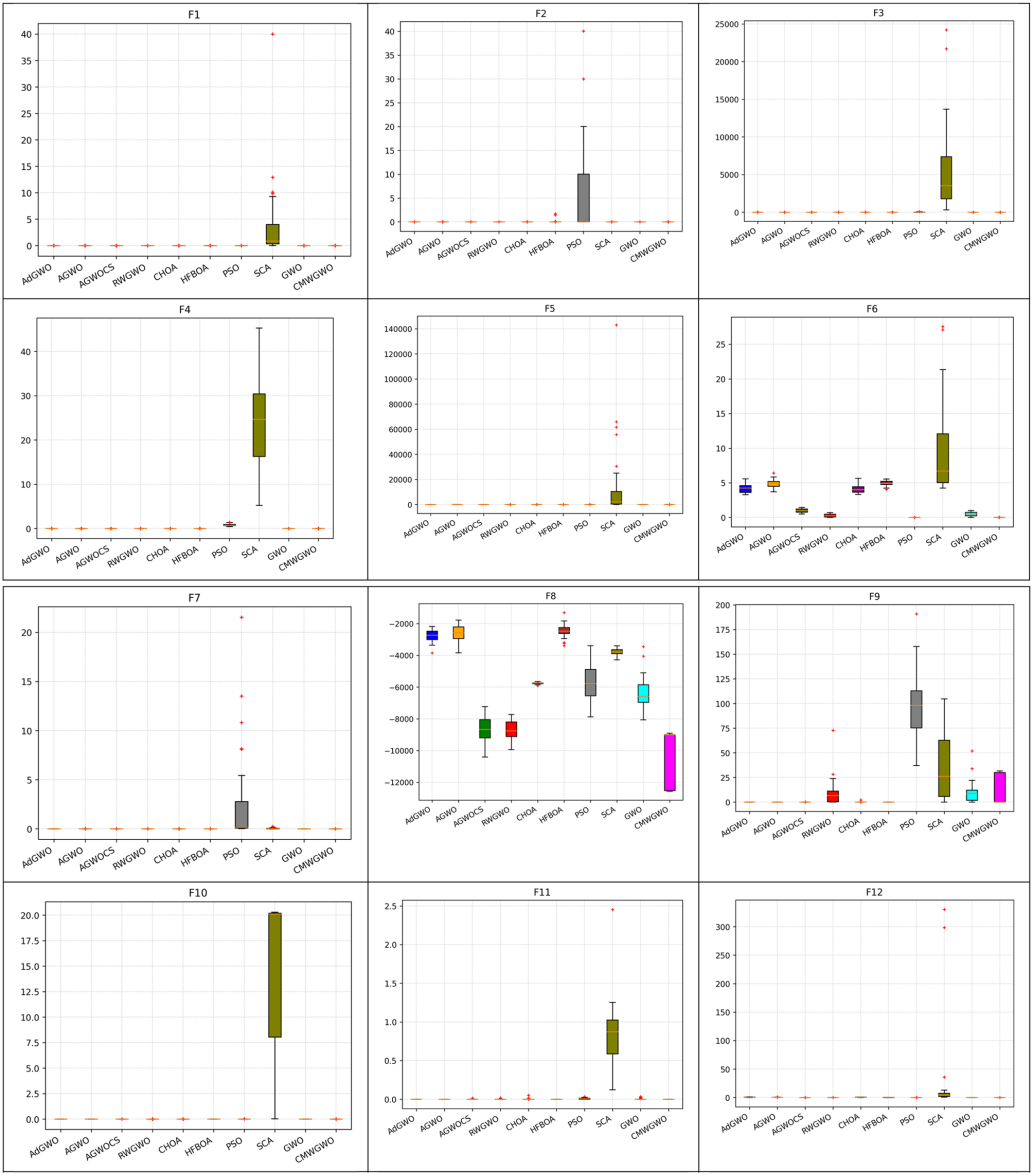

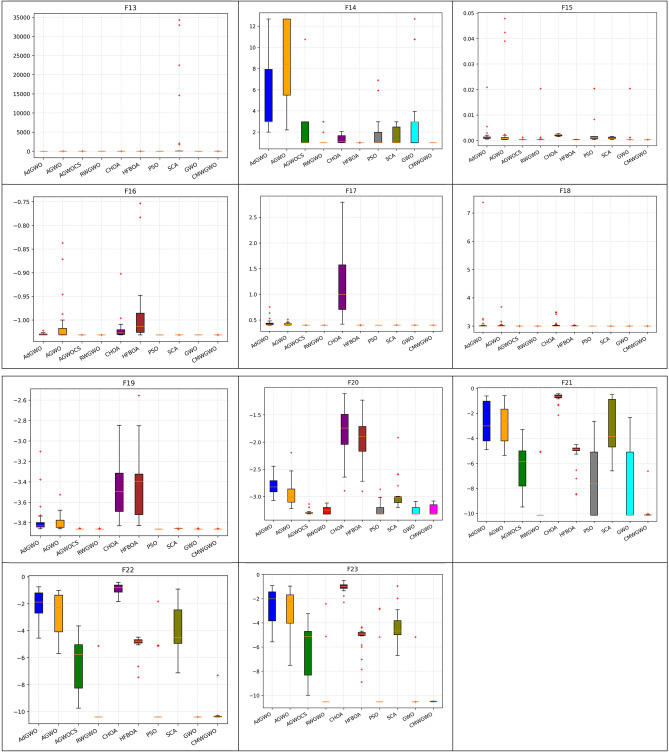
Box plot of CMWGWO and nine compared optimizers on 23 functions.

**Figure 12:**
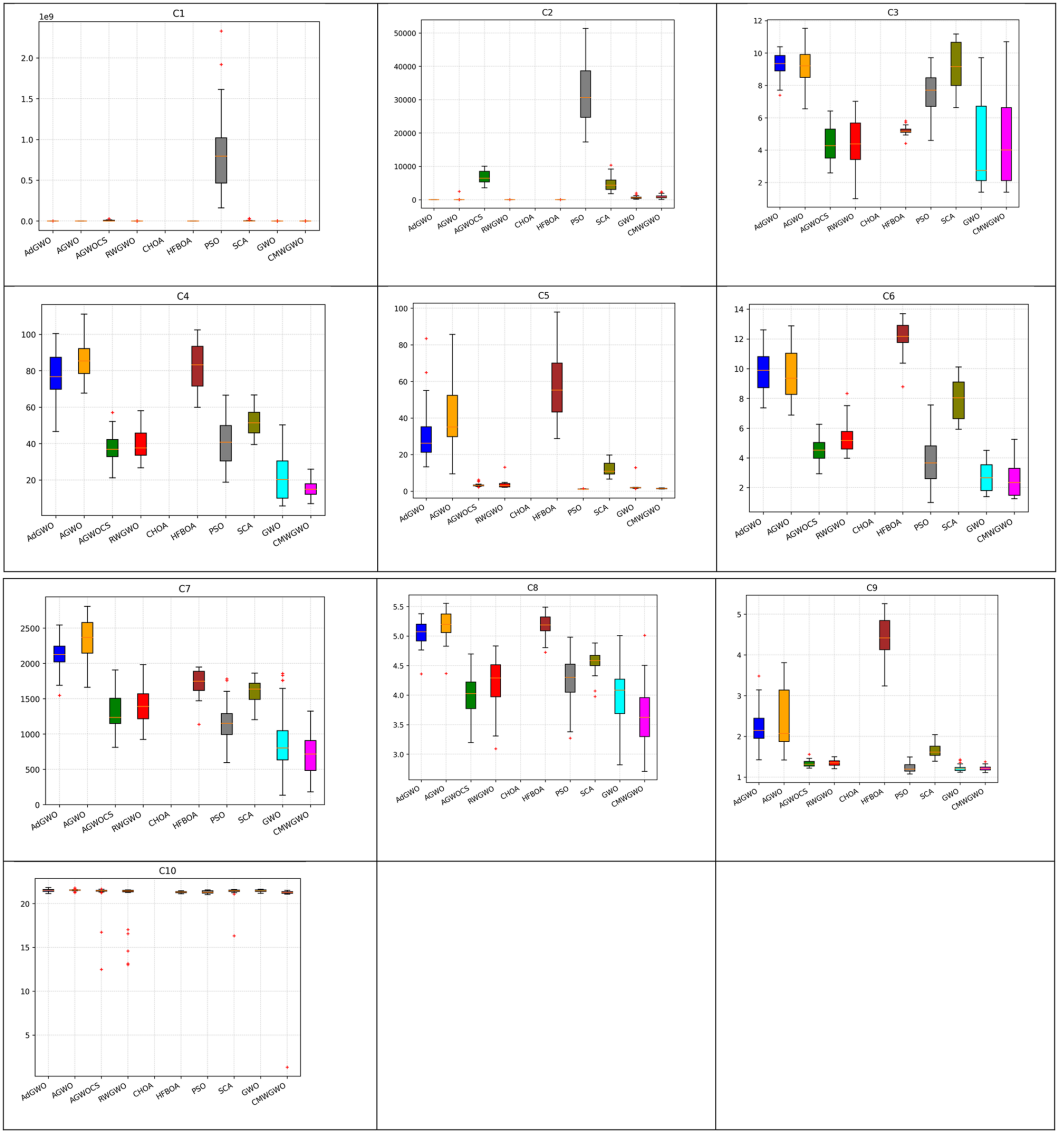
Box plot of CMWGWO and nine compared optimizers on CEC 2019 functions.

Figures 9, 10, 11 and 12 compare the CMWGWO method with nine different cutting-edge algorithms using convergence curves and box plots on 23 functions (30 dim) and CEC 2019 respectively. These charts show how each algorithm's average accuracy changes as the number of iterations rises, as shown in Figs. 9 and 10. The distribution of the final optimal solutions attained by each method is shown by the box plots. The minimum, maximum, lower quartile (Q1), median, upper quartile (Q3), and any outliers can all be viewed clearly inside the box plots in Figs. 11 and 12. The best set of solutions from each iteration of 30 iterations is displayed by a box plot, while the orange line inside the box denotes the median. Notably, an outlier is a data point that deviates significantly from the norm and is identified by a red “+” sign. This comparison's goal is to illustrate and assess the variations in optimization performance between CMWGWO and other cutting-edge algorithms. The convergence curves provide information on how the best solution values change when the search process of each approach is performed. A low value representing the best solution found indicates that the approach is more capable of optimization. The box plots, on the other hand, give details about how the best results from each approach are distributed. A technique is more stable and hence more resistant to changes in the search space if the approach's box sizes in the box plots are smaller. To put it another way, the box plots illustrate how consistently each approach finds the ideal answer while the convergence curves show how effectively each method achieves that goal.

The CMWGWO technique displays quick convergence in its early phases, as seen in Fig. 9. It's interesting to note that the CMWGWO approach continues to explore high-quality regions while other algorithms tend to have a flattened curve meaning they can easily be stuck in local optimal. Furthermore, according to the findings, CMWGWO demonstrates quicker convergence for all functions other than F7 in uni-modal functions (F1-F7). The suggested technique, however, performs better for multimodal functions than current approaches, with better results for functions F8, F11, F12, and F13. Additionally, the suggested method exhibits admirable and exceptional convergence for functions F14–F19, F21, and F23, categorized as fixed-dimension functions. Notably, the CMWGWO outperforms AGWO, AdGWO, and AGWOCS in establishing a balance between convergence and divergence. Figure 9’s comparison further demonstrates that CMWGWO maintains higher convergence accuracy than other techniques. These findings confirm that, in comparison to the traditional GWO approach, the modifications made in this work not only improve the trade-off between exploration and exploitation, it also demonstrate the method's capacity to avoid local optima and get close to the overall best outcome. Three crucial strategies WID, COL, and MRS were incorporated into the CMWGWO technique to increase its effectiveness in this area. While the COL technique increases population variation throughout the search process, the MRS strategy enables the wolf agent to keep investigating the optimum solution. The WID tactic also effectively traps prey, all these add to the efficiency of CMWGWO. Furthermore, the CMWGWO approach is able to find probable solutions inside the problem domain characterized by shifted, rotated, and hybrid in CEC 2019 functions in Fig. 10 because of the combination of various tactics, which finally results in improved diversity and more accurate solutions in functions C1, C4, C6, C7, C8, and C9. The boxplot analysis of each function also makes it quite evident that CMWGWO has strong stability as seen in Figs. 11 and 12. This suggests that the CMWGWO’s approach to exploration and exploitation capabilities is well-balanced.

### Exploration and exploitation analysis

Exploration and exploitation stages are often two essential phases in optimization algorithms. The Algorithm prioritizes exploration in the first stage with the goal of identifying areas of the feasible domain space that have promising prospects for improved candidate solutions. The algorithm then progressively moves from the exploration to the exploitation , putting more effort into looking for better candidate solutions close to the existing best solution. An algorithm's optimization efficiency is largely influenced by how well its exploration and exploitation capabilities are balanced. The chances of discovering improved candidate solutions may increase with more exploration capabilities, but the speed of convergence may be slowed. On the other hand, increasing the exploitation capabilities might hasten convergence but increase the chance of being stuck in local optima. To establish a delicate balance between the exploration and exploitation phases, we enhanced CMWGWO’s exploitation and exploration. This balance is essential since it affects the effectiveness of optimization as a whole. In order to locate high-quality solutions quickly while avoiding premature convergence to local optima, The algorithm must ideally balance exploration and exploitation. To enhance the algorithm's efficacy and resilience in tackling optimization issues.

In this part, the exploration and exploitation stages of the CMWGWO are numerically investigated and compared to the traditional GWO. We use Eqs. (20) to (23) to determine the proportion of these two phases in order to more accurately characterize the algorithm's exploration and exploitation process while it is running.

20 $${\text{\% }}EPR = \frac{Div}{{Div_{max} }} \times 100$$

21 $${\text{\% }}EPL = \frac{{\left| {Div - Div_{max} } \right|}}{{D_{{iv_{max} }} }} \times 100$$

22 $${\text{Div}}_{j} = \frac{1}{n}\mathop \sum \limits_{i = 1}^{n} \left| {{\text{median}}\left( {x^{j} } \right) - x_{i}^{j} } \right|$$

23 $$Div = \frac{1}{{{\text{dim}}}}\mathop \sum \limits_{j = 1}^{{{\text{dim}}}} Div_{j}$$

The percentages of the algorithm's exploration and exploitation stages are shown by the symbols ${\text{\% }}EPR$ and ${\text{\% }}EPL$, respectively. The diversity of all population members in the technique is denoted by $Div$ and $Div_{max}$ denotes the highest diversity value thus far observed among the population members. Furthermore, $Div_{j}$ stands for the diversity of the $jth$ dimension throughout the whole population. The algorithm's parameters $n$ and $dim$ correspond to the population's size and the problem's dimension, respectively. While ${\text{median}}\left( {x^{j} } \right)$ designates the median value of the $jth$ dimension across all population members, $x^{j}$ specifies the $jth$ dimension of the $ith$ member in the technique.

**Figure 13:**
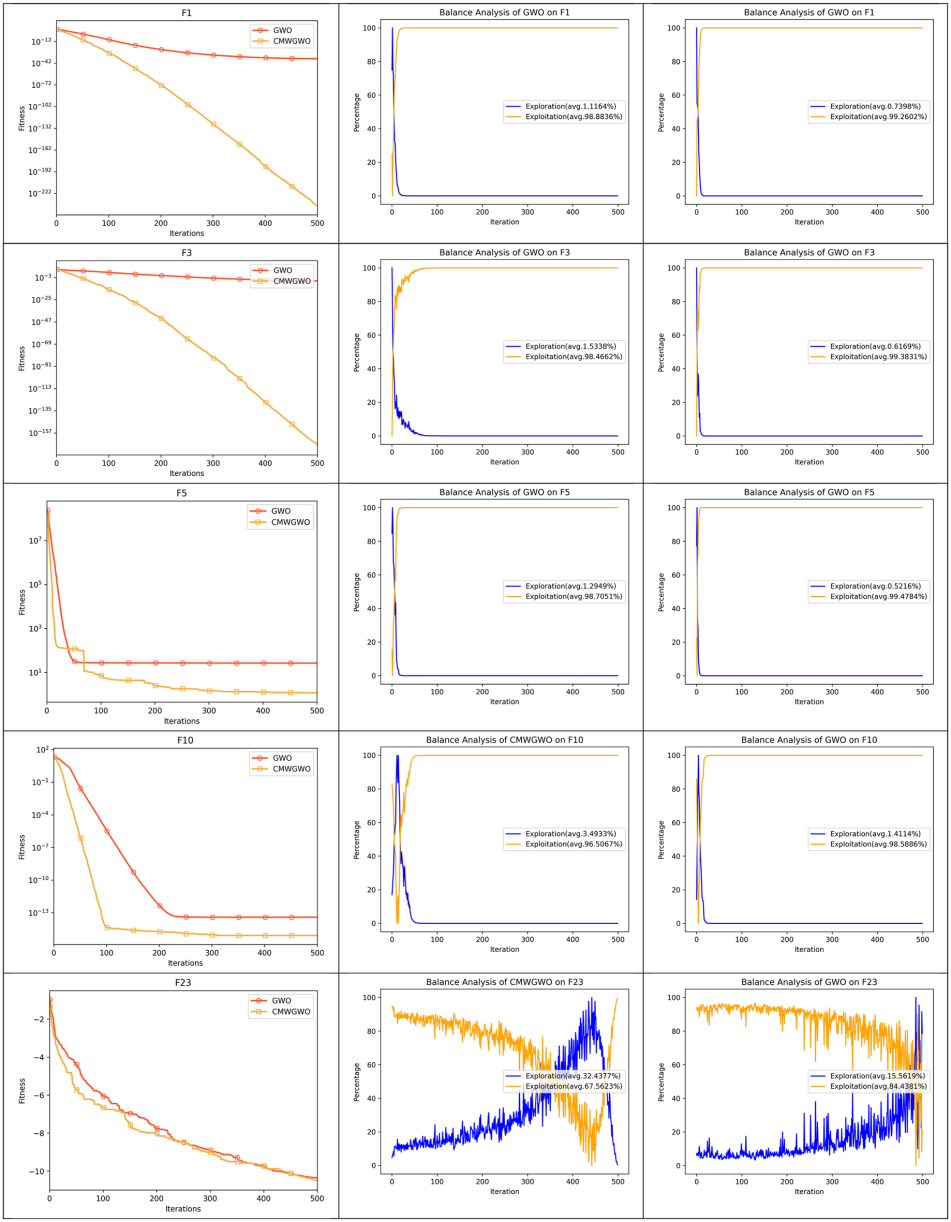
Exploration and exploitation comparison of CMWGWO and GWO.

Specific illustrations depicting unimodal and multimodal functions selected from the functions utilized in the previous experiment are used to analyze the algorithm's exploration and exploitation levels during their search process, as shown in Fig. 13. The first column compares the convergence curves of CMWGWO and GWO, while the second and third columns show the exploration and exploitation phases of CMWGWO and GWO, respectively. F1, F3, and F5 are categorized as unimodal functions, whereas F10 and F23 are categorized as multimodal. The balance of the suggested CMGWO and GWO for unimodal and multimodal functions is shown in Fig. 13 by the convergence and diversity patterns. It is clear that when compared to the original GWO approach, the CMWGWO method shows enhanced exploration of optimum solutions. Additionally, CMWGWO outperforms GWO in terms of striking a balance between the algorithm's exploitation and exploration stages.

The percentage of exploration length (${\text{\% }}EPR)$ attained by the CMWGWO approach is as follows when looking at the second column of Fig. 13: 1.1164% for F1, 1.5338% for F3, 1.2949% for F5, 3.4933% for F10, and 32.4377% for F23. Furthermore the ${\text{\% }}EPL$ is 98.8836% for F1, 98.4662% for F3, 98.7051% for F5, 96.5067% for F10, and 67.5623% for F23. The suggested CMWGWO approach exhibits an increase of around 2.1% on the unimodal functions F1, F3, and F5 in the exploration phase when compared to the ${\text{\% }}EPR$ attained by GWO in the unimodal functions. Additionally, there is an increase of around 19% in the exploration phase compared to GWO for the multimodal functions F10 and F23. It can be concluded that the proposed CMWGWO more efficiently divides the execution time between the exploitation and exploration phases of the algorithm based on the convergence curves of CMWGWO and GWO on F1, F3, F5, F10, and F23. To put it another way, it shows a greater balance between the two stages, which enhances performance.

### Computation time analysis

**Figure 14:**
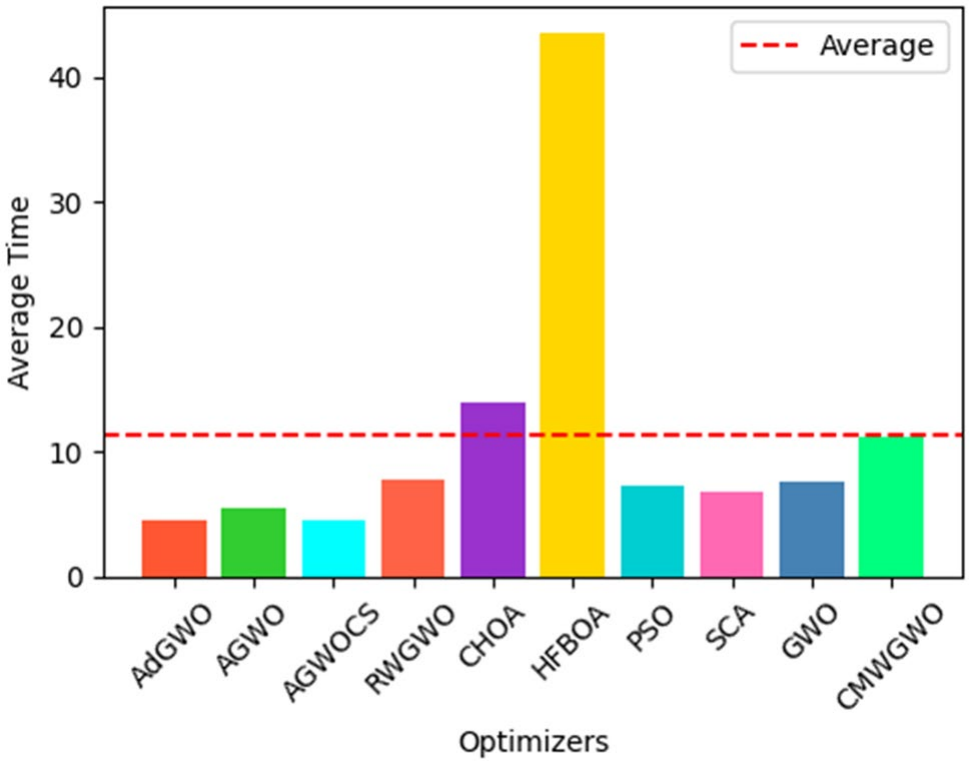
Comparison of optimizer average computation time on 23 functions.

**Figure 15:**
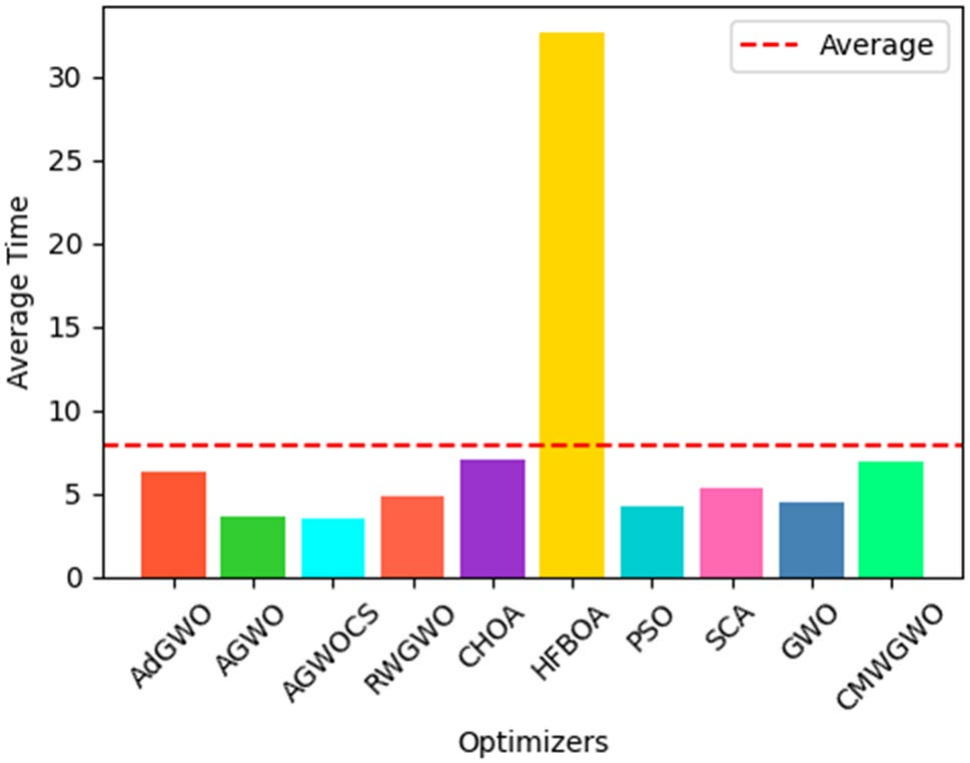
Comparison of optimizer average computation time on CEC 2019 functions.

Tables 10 and 11 present a comparison of the average computation time of CMWGWO and its competitors. A detailed analysis of CMWGWO highlights that it generally necessitates more CPU time when compared to other methods. This can be attributed to CMWGWO's incorporation of MRS, COL, and WID, wherein each method is independently executed in the course of the optimization process. Consequently, the CPU time of CMWGWO does not consistently outperform the compared methods due to its inherent complexity, as elucidated in Eq. 19. In Figs. 14 and 15, it becomes evident that CMWGWO requires greater computational time than the original GWO and other GWO variants such as AdGWO, AGWO, AGWOCS, and RWGWO. Nonetheless, despite its increased computational demands, CMWGWO exhibits remarkable efficiency, surpassing these algorithms in terms of performance. Taking into consideration the substantial contributions of CMWGWO, a harmonious balance can be achieved between attaining high accuracy and effectively managing the time required to solve problems.

**Table 10:** Computation time comparison of CMWGWO with GWO variants and original algorithms on 23 functions.

	AdGWO	AGWO	AGWOCS	RWGWO	CHOA	HFBOA	PSO	SCA	GWO	CMWGWO
F1	2.929	3.449	**2.76**	10.845	18.835	15.471	9.423	9.347	10.195	14.85
F2	**3.649**	5.497	4.133	10.832	20.889	17.563	9.599	9.357	10.808	16.247
F3	8.074	8.882	**7.493**	13.242	23.149	75.456	11.85	11.56	13.006	20.253
F4	5.651	16.02	**5.101**	10.672	20.812	14.597	9.431	9.187	10.619	6.489
F5	5.82	6.657	**5.255**	10.893	20.91	19.381	9.618	9.393	10.834	16.361
F6	5.721	6.538	**5.162**	10.769	20.857	16.815	16.136	9.267	10.761	9.547
F7	5.895	6.708	**5.252**	10.507	21.024	21.457	9.268	8.996	10.44	15.761
F8	5.733	6.547	**5.175**	10.792	20.896	16.059	9.659	9.294	10.71	16.211
F9	5.746	6.565	**5.094**	10.315	20.709	16.291	9.042	8.773	10.286	15.452
10	5.909	6.741	**5.318**	10.937	20.774	21.203	9.789	9.435	10.882	16.456
F11	5.964	6.779	**5.371**	10.993	20.853	17.185	9.837	9.498	10.936	16.499
F12	6.554	7.371	**5.954**	11.671	21.627	37.714	10.427	10.121	11.54	17.39
F13	6.819	7.648	**6.137**	11.972	22.142	42.986	10.616	10.314	11.863	17.8
F14	8.204	7.991	8.596	8.544	9.199	200.195	**7.942**	7.968	8.116	14.072
F15	**1.545**	1.554	2.033	1.905	3.537	22.842	1.692	1.645	1.844	2.959
F16	0.86	0.798	1.45	0.8	1.825	13.688	0.703	**0.677**	0.783	1.314
F17	0.88	0.799	1.472	0.822	1.841	14.02	0.717	**0.687**	0.796	1.33
F18	0.92	0.84	1.514	0.862	1.879	15.245	0.759	**0.735**	0.838	1.425
F19	1.722	1.702	2.273	1.944	3.204	31.981	1.731	**1.7**	1.882	3.151
F20	**2.271**	2.37	2.674	3.041	5.277	33.054	2.73	2.661	2.986	4.794
F21	**3.691**	3.749	4.195	4.437	5.934	82.535	3.862	4.052	4.222	7.302
F22	**4.62**	4.652	5.126	5.525	6.936	108.332	4.778	5.05	5.193	8.995
F23	**6.013**	6.05	6.539	7.007	8.429	146.07	6.178	6.559	6.688	11.705
TOTAL AVG	4.573478	5.474217	**4.525087**	7.796826	13.97991	43.48435	7.20813	6.794609	7.662087	11.14622

Significant values are in [bold].

**Table 11:** Computation time comparison of CMWGWO with GWO variants and original algorithms on CEC 2019.

	AdGWO	AGWO	AGWOCS	RWGWO	CHOA	HFBOA	PSO	SCA	GWO	CMWGWO
C1	5.877	**1.858**	1.913	2.519	4.851	10.600	2.215	4.187	2.365	3.687
C2	8.998	2.919	**2.641**	4.226	8.276	10.581	3.694	4.857	3.998	5.983
C3	9.795	3.308	**2.765**	4.716	9.147	10.634	4.038	9.209	4.544	6.722
C4	12.691	3.998	**3.762**	5.055	6.863	41.514	4.495	5.717	4.688	7.425
C5	5.906	4.043	**3.729**	5.060	6.840	41.247	4.473	12.136	4.733	7.371
C6	**4.159**	4.435	4.278	5.569	7.221	47.230	4.900	7.877	5.154	7.995
C7	3.898	4.068	4.026	5.322	6.841	41.479	4.564	**1.375**	4.792	7.364
C8	**3.915**	3.944	4.017	5.301	6.845	41.204	4.679	4.572	4.856	7.383
C9	3.941	3.927	4.030	5.254	6.862	40.863	4.669	**1.639**	4.884	7.302
C10	3.914	4.096	4.039	4.910	6.874	41.027	4.705	**2.285**	4.898	7.416
Total Avg	6.309	3.660	**3.520**	4.793	7.062	32.638	4.243	5.385	4.491	6.865

Significant values are in [bold].

### Engineering problem application

Based on the constraints and particular needs of the optimization method they are employing, researchers must take thorough and well-founded assessments. They need efficient tools that provide them the ability to make wise decisions within a logical framework in order to do this ^71,72^. By using it to solve three traditional engineering constraint issues, the performance of CMWGWO is carefully assessed in this context. The purpose of this inquiry is to confirm the useful and practical uses of the CMWGWO approach. The three issues under consideration are as follows: Welded Beam Design Problem (WBDP)^73^, Three Truss Bar (TTB)^74,75^ and I-Beam Design Problem(IBDP)^76,77^.

#### Welded beam design (WBDP)

**Figure 16:**
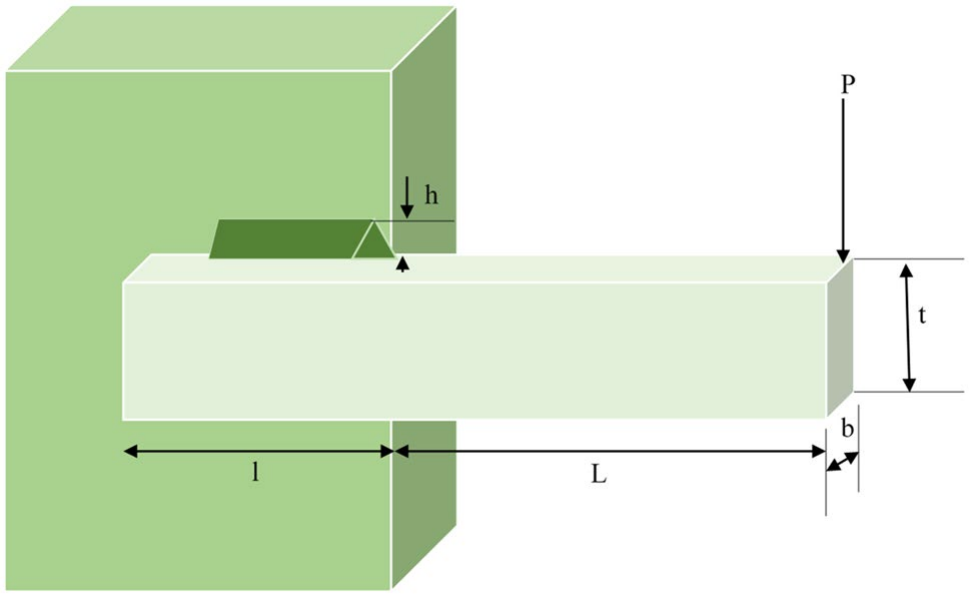
Welded beam design problem.

In the welded beam problem, a stiff support member needs to be welded to a beam. The ideal cost problem, depicted in Fig. 16, is used to estimate the beam's ideal dimensions in order to reduce costs^78^. Four main factors, namely, weld seam thickness (h(${\text{x}}_{1} )$), steel bar length (l $\left( {{\text{x}}_{2} } \right)$), steel bar height (t $\left( {{\text{x}}_{3} } \right)$) and steel bar thickness (b $\left( {{\text{x}}_{4} } \right)$), have an impact on the production cost. Additionally, the model is subject to four constraints: buckling load (Pc), shear stress (*τ*), beam internal bending stress (*σ*), and end deflection rate (δ). The mathematical expression of this problem can be stated as in Fig. 16.

Objective function

24 $${\text{F}}\left( {\text{X}} \right) = 1.10471{\text{x}}_{1}^{2} {\text{x}}_{2} + 0.04811{\text{x}}_{3} {\text{x}}_{4} \left( {14.0 + {\text{x}}_{2} } \right).$$

Subject to:

25 $${\text{g}}_{1} \left( {\text{X}} \right) = \tau \left( {\text{X}} \right) - \tau_{{{\text{max}}}} \le 0$$

26 $${\text{ g}}_{2} \left( {\text{X}} \right) = \sigma \left( {\text{X}} \right) - \sigma_{{{\text{max}}}} \le 0$$

27 $${\text{ g}}_{3} \left( {\text{X}} \right) = {\text{x}}_{1} - {\text{x}}_{4} \le 0$$

28 $${\text{ g}}_{4} \left( {\text{X}} \right) = 0.10471{\text{x}}^{2} _{1} + 0.04811{\text{x}}_{3} {\text{x}}_{4} \left( {14.0 + {\text{x}}_{2} } \right) - 5.0 \le 0$$

29 $${\text{ g}}_{5} \left( {\text{X}} \right) = 0.125 - {\text{x}}_{1} \le 0$$

30 $${\text{ g}}_{6} \left( {\text{X}} \right) = \delta \left( {\text{X}} \right) - \delta_{{{\text{max}}}} \le 0$$

31 $${\text{ g}}_{7} \left( {\text{X}} \right) = {\text{P}} - {\text{P}}_{{\text{c}}} \left( {\text{X}} \right) \le 0$$

where

32 $$\tau \left( {\text{X}} \right) = \sqrt {\left( {\tau^{\prime } } \right)^{2} + 2\tau^{\prime } \tau^{\prime \prime } \frac{{{\text{x}}_{2} }}{{2{\text{R}}}} + \left( {\tau^{\prime \prime } } \right)^{2} }$$

33 $$\tau^{\prime } = \frac{{\text{P}}}{{\sqrt 2 {\text{x}}_{1} {\text{x}}_{2} }},\tau^{\prime \prime } = \frac{{{\text{MR}}}}{{\text{J}}},{\text{M}} = {\text{P}}\left( {{\text{L}} + \frac{{{\text{x}}_{2} }}{2}} \right)$$

34 $${\text{R}} = \sqrt {\frac{{x_{2}^{2} }}{4}{ } + \left( {\frac{{{\text{x}}_{1} + {\text{x}}_{3} }}{2}} \right)^{2} }$$

35 $${\text{J}} = 2\left\{ {\sqrt 2 {\text{x}}_{1} {\text{x}}_{2} \left[ {\frac{{{\text{x}}_{2}^{2} }}{12} + { }\left( {\frac{{{\text{x}}_{1} + {\text{x}}_{3} }}{2}} \right)^{2} } \right]} \right\}$$

36 $$\sigma \left( {\text{X}} \right) = \frac{6PL}{{{\text{x}}_{4} {\text{x}}_{3}^{2} }},\delta \left( {\text{X}} \right) = \frac{{4PL^{3} }}{{{\text{Ex}}_{3}^{3} {\text{x}}_{4} }}$$

37 $${\text{P}}_{{\text{c}}} \left( {\text{X}} \right) = \frac{{4.013E\sqrt {\frac{{{\text{x}}_{3}^{2} {\text{x}}_{4}^{6} }}{36}} }}{{{\text{ L}}^{2} }}\left( {1 - \frac{{{\text{x}}_{3} }}{2L}\sqrt{\frac{E}{4G}} } \right)$$

38 $${\text{P}} = 6000\;{\text{lb}},\;{\text{L}} = 14\;{\text{in}},\;{\text{E}} = 30 \times 10^{6} \;{\text{psi}},\;{\text{G}} = 12 \times 10^{6} \;{\text{psi}}$$

39 $$\tau_{{{\text{max}}}} = 13,600\;{\text{psi}},\;\sigma_{{{\text{max}}}} = 30,000\;{\text{psi}},\;\delta_{{{\text{max}}}} = 0.25\;{\text{in}}{.}$$

Based on the data shown in Table 12, the results reveal that the CMWGWO method attains the smallest cost for WBDP, measuring 1.670217726. This outcome highlights a significant advantage over the GWO, RWGWO, and AGWOCS algorithms. Clearly, CMWGWO effectively meets the requirements of the design problem with the lowest cost, leading to reduced engineering consumption. These findings demonstrate the practical superiority of CMWGWO in achieving optimal solutions, resulting in cost-effective designs and resource savings in engineering applications.

**Table 12:** Results of CMWGWO and other algorithms on WBDP.

	Optimal Cost	h	l	t	b
CMWGWO	**1.670217726**	0.198832	3.337365	9.192024	0.198832
GWO	1.670556251	0.198805	3.338965	9.191231	0.198871
SCA	1.70353017	0.199921	3.290179	9.359652	0.200145
RWGWO	1.670846301	0.198712	3.339064	9.196423	0.198813
PSO	1.674171966	0.198372	3.347039	9.215288	0.198767
AGWOCS	1.690831828	0.192598	3.491132	9.200901	0.199905
AdGWO	1.692837585	0.204689	3.266280	9.065060	0.204561
AGWO	1.672764540	0.197565	3.363120	9.194295	0.198916
CHOA	1.726704789	0.194029	3.430487	9.522930	0.198357
HFBOA	1.708626666	0.198331	3.522627	9.224930	0.200026

Significant values are in [bold].

#### Three truss bar (TTB)

**Figure 17:**
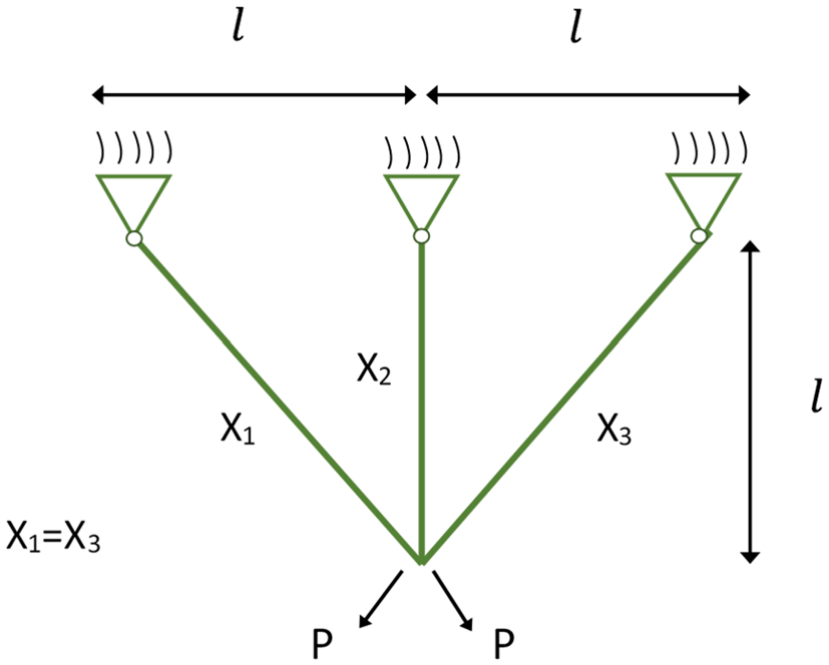
Three bar truss design problem.

Firstly introduced by Ray and Saini, the three bar truss design optimization problem is a classic engineering optimization problem in structural mechanics^79^. The problem consists of two variables and three constraints. It involves finding the optimal dimensions of a truss made of three bars to achieve certain design objectives while respecting constraints such as buckling, stress, and bending, as presented in Fig. 17.

Objective function:

40 $$f\left( {x_{1} ,x_{2} } \right) = l \times \left( {2\sqrt 2 x_{1} + x_{2} } \right).$$

Subject to by:

41 $$G_{1} = \frac{{\sqrt 2 x_{1} + x_{2} }}{{\sqrt 2 x_{1} 2 + 2x_{1} x_{2} }}P - \sigma \le 0$$

42 $$G_{2} = \frac{{x_{2} }}{{\sqrt 2 x_{1} 2 + 2x_{1} x_{2} }}P - \sigma \le 0$$

43 $$G_{3} = \frac{1}{{\sqrt 2 x_{2} + x_{1} }}P - \sigma \le 0$$

where $$l = 100\;{\text{cm}};\;P = \frac{{2\;{\text{kN}}}}{{{\text{cm}}^{2} }};\;\sigma = \frac{{2\;{\text{kN}}}}{{{\text{cm}}^{2} }}$$.

The information in Table 13 makes it readily apparent that the CMWGWO approach earns the top spot in terms of best costs. This result shows that the CMWGWO, works remarkably well for this particular situation. It verifies the suggested algorithm's superiority over competing approaches and shows that it can produce cost-optimization solutions that are both highly competitive and superior.

**Table 13:** Results of CMWGWO and other algorithms on TTB.

	Optimal weight	X_1_	X_2_
CMWGWO	**263.8958434**	0.788581	0.408515
GWO	263.8959482	0.788386	0.409066
SCA	263.9022373	0.787461	0.411747
RWGWO	263.8959996	0.788735	0.40808
PSO	263.8964742	0.789473	0.405999
AGWOCS	263.8984792	0.788959	0.407473
AdGWO	264.0293293	0.776680	0.443508
AGWO	263.8978898	0.789184	0.406827
CHOA	263.9262500	0.786991	0.413315
HFBOA	263.8980334	0.790019	0.404466

Significant values are in [bold].

#### I-beam design problem (IBDP)

**Figure 18:**
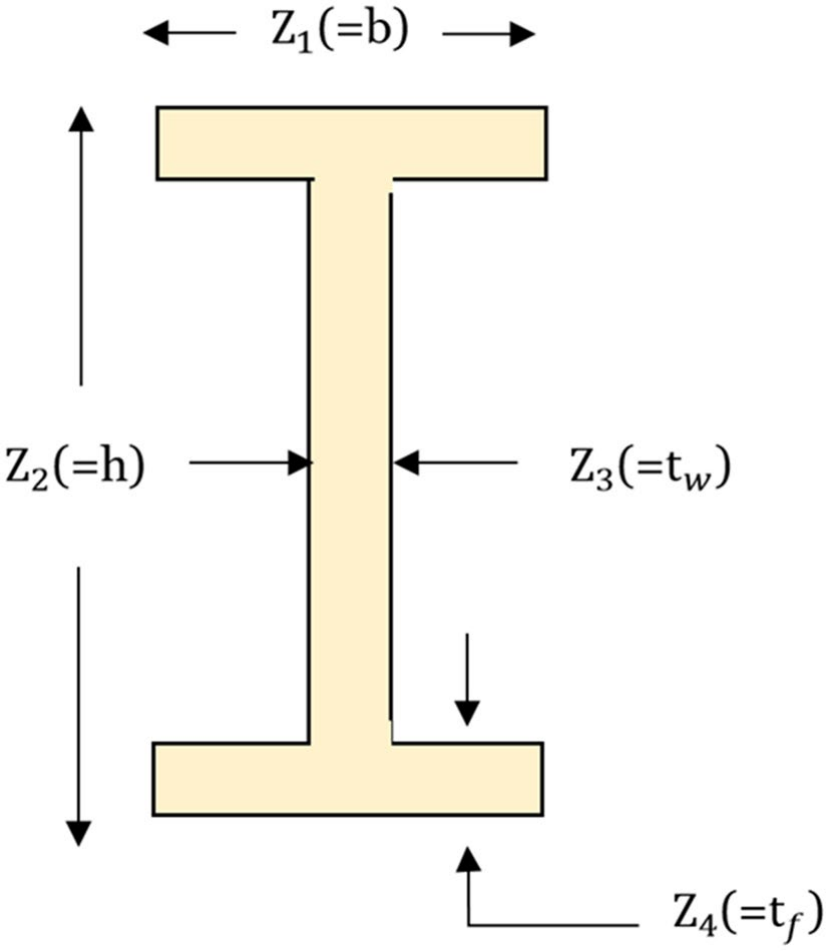
I-beam design problem.

The I-beam design problem, as shown in Fig. 18, involves a beam subjected to two pressures^80^. The goal is to design an I-beam with minimal vertical deflection. The structural parameters of the problem consist of height, length, and two thicknesses. The mathematical representation of this problem is presented below:

Objective function:

44 $$f\left( z \right) = \frac{5000}{{\frac{{z_{3} \times \left( {z_{2} - 2z_{4} } \right)^{3} }}{12} + \left( {\frac{{z_{1} \times z_{4}^{3} }}{6}} \right) + 2b \times z_{4} \left( {z_{2} - \frac{{z_{4} }}{2}} \right)^{2} }}.$$

Subject to:

45 $$\begin{aligned} & g_{1} \left( z \right) = 2z_{1} \times z_{3} + z_{3} \times \left( {z_{2} - 2z_{4} } \right) \le 300, \\ & g_{2} \left( z \right) = \frac{{18z_{2} \times 10^{4} }}{{z_{3} \left( {z_{2} - 2z_{4} } \right)^{3} + 2z_{1} z_{3} \left( {4z_{4}^{2} + 3z_{2} \left( {z_{2} - 2z_{4} } \right)} \right)}} + \frac{{15z_{1} \times 10^{3} }}{{z_{3}^{2} \left( {z_{2} - 2z_{4} } \right) + 2z_{3} z_{1}^{3} }} \le 56, \\ \end{aligned}$$

where $$10 \le z_{1} \le 50$$, $$10 \le z_{2} \le 80$$, $$0.9 \le z_{3} ,z_{4} \le 5$$.

The CMWGWO is compared to a number of optimization techniques as seen in Table 14, Table 14 displays the experimental results. It is evident from observing the data that CMWGWO obtains the smallest vertical deflection, measuring 0.013074119. This outstanding outcome demonstrates that, when compared to other optimization techniques, CMWGWO provides the best answer for this particular problem design type.

**Table 14:** Results of CMWGWO and other algorithms on IBDP.

	Optimal deflection	Z_1_	Z_2_	Z_3_	Z_4_
CMWGWO	**0.013074119**	80	50	0.9	2.321792
GWO	0.013074134	80	50	0.9	2.321789
SCA	0.013074296	80	50	0.9	2.321755
RWGWO	0.013074159	80	50	0.9	2.321783
PSO	0.01307413	80	50	0.9	2.32179
AGWOCS	0.013074337	80	50	0.9	2.321746
AdGWO	0.013080006	80	50	0.9	2.320557
AGWO	0.013074418	80	50	0.9	2.321729
CHOA	0.013077711	80	50	0.9	2.321038
HFBOA	0.013077141	80	50	0.9	2.321158

Significant values are in [bold].

## Conclusion

This paper introduces CMWGWO with the primary objective of addressing the limitations of the original GWO. These limitations include premature convergence, insufficient diversity within the population, subpar global search capabilities, and susceptibility to be trapped in local optimum due to convergence towards the best wolf. CMWGWO employs three strategies to overcome these limitations. Firstly, the WID strategy is employed to enhance population diversity by facilitating better information exchange between the best and worst wolves. This improvement enables the algorithm to escape stagnation and explore a more extensive range of solutions. Secondly, the algorithm incorporates the embedded COL search mechanism to increase the likelihood of individuals approaching the global optimum. By doing so, it elevates the optimization accuracy and alleviates stagnation issues. Lastly, the integration of MRS amplifies population exploration and significantly expands the search space. As a result, CMWGWO is able to effectively explore a wider range of potential solutions, enhancing its overall performance in optimization tasks.

The experiments in this study involve the testing of 23 functions and 10 CEC 2019 with distinct characteristics. The initial comparison includes WID_GWO, COL_GWO, MRS_GWO, GWO, and CMWGWO to confirm the effectiveness of the optimization mechanisms introduced in this paper. Furthermore, CMWGWO is pitted against well-known GWO variants, namely RWGWO, AGWO, AdGWO, and AGWOCS. The results clearly demonstrate that CMWGWO outperforms these competitive algorithms significantly, a fact that becomes evident when examining the convergence curves of these algorithms. In contrast to the original algorithms, such as CHOA, SCA, HFBOA, and PSO, CMWGWO exhibits a robust exploration ability and improves solution accuracy substantially. Extensive testing on high-dimensional problems, coupled with exploitation and diversity analysis, further confirms its capability to achieve higher-quality solutions. Lastly, the application of CMWGWO to WBDP, TTB, and IBDP problems showcases its effectiveness in effectively solving these typical engineering constraint problems, thereby highlighting its potential for practical applications.

Although CMWGWO can surpass the original GWO and other rival algorithms, its optimization performance can yet be enhanced. Tables 5, 6, 7 and 9 display the results of such functions i.e. F7 and F9 functions. This proves the No Free Lunch theorem that no single optimizer is efficient for all problems. To attain greater solution accuracy, we intend to improve CMWGWO's exploration and exploitation capabilities going forward. This will need combining more modification approaches, such as applying novel population initializing strategies, hybridizing with other algorithms, and adaptively lowering some parameters in a nonlinear way. Additionally, CMWGWO has difficulties when tackling large-scale and complicated issues; therefore, future work will entail extensive tests on complex problems and comparison with more state-of-the-art algorithms. CMWGWO requires more time than the original GWO, making it necessary to take into account parallel computing in the next research stage to speed up the procedure. A fascinating research path also involves merging CMWGWO with machine learning. Furthermore, the applicability of CMWGWO can be extended to various real-world optimization problems across different fields. For instance, it can be effectively utilized in optimal power flow problems ^81^, classification of neuroimaging^82^, heat removal systems^83^, and water distribution systems^84^. Expanding CMWGWO’s potential, it would be reasonable to explore the development of a multi-objective version of the algorithm, catering to complex multi-objective challenges that require simultaneous optimization of multiple criteria.

## Data Availability

The data obtained through the experiments are available upon request from the corresponding author.
